# Pyrazine‐Functionalized Ru(II)‐Complexes as Visible‐Light Activated Photobases

**DOI:** 10.1002/chem.202404033

**Published:** 2025-04-28

**Authors:** Niklas Klosterhalfen, Nishi Singh, Michael Jäger, Andreas Winter, Phil Köhler, Ulrich S. Schubert, Benjamin Dietzek‐Ivanšić

**Affiliations:** ^1^ Department Functional Interfaces Leibniz Institute of Photonic Technology (Leibniz‐IPHT) Albert‐Einstein‐Str. 9 Jena Germany; ^2^ Institute for Physical Chemistry (IPC) Friedrich Schiller University Jena Helmholtzweg 4 Jena Germany; ^3^ Laboratory of Organic and Macromolecular Chemistry (IOMC) Friedrich Schiller University Jena Humboldtstr. 10 Jena Germany; ^4^ Center for Energy and Environmental Chemistry Jena (CEEC Jena) Friedrich Schiller University Jena Philosophenweg 7a Jena Germany; ^5^ Institute for Inorganic and Analytical Chemistry (IAAC) Friedrich Schiller University Jena Humboldtstr. 8 Jena Germany

**Keywords:** excited‐state proton transfer, Ru(II) complexes as photobases, synthesis and characterization, photophysical and theoretical studies, ultrafast spectroscopy

## Abstract

Excited‐state proton transfer (ESPT) roots in significantly increased acidities or basicities of up to ca. 10 p*K*_a_ units in the excited state compared to the ground state. While organic photobases are either of “single use” in the case of photobase generators or are limited functionally by their UV absorption, metal complex‐based photobases offer intriguing properties. By exciting the characteristic metal‐to‐ligand charge transfer (MLCT) transitions in the visible range, multiple and reversible ESPT processes can be triggered. In this contribution we present the synthesis of two novel Ruthenium(II)‐complexes with pyrazine‐functionalized polypyridyl ligands and study their photobasic properties by ultrafast spectroscopy. We find that MLCT excitation in aqueous solution leads to a Δp*K*_a_ of 9 units and that the involved ESPT process takes place within ca. 300 picoseconds. Our investigations combine experimental spectroscopy with theoretical calculations.

## Graphical abstract

Visible light excitation leads to the accumulation of electron density in pyrazine‐containing Ru(II) complexes via metal‐to‐ligand charge transfer (MLCT). In aqueous solutions of appropriate pH, excited‐state proton transfer (ESPT) takes place on a timescale of hundreds of ps. We resolve the underlying kinetics via transient absorption (TA) spectroscopy and corroborate our studies by photometric titrations and DFT calculations.

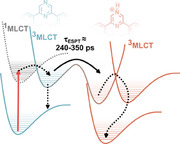

## 1 Introduction

Proton transfer reactions play a key role in chemistry, material sciences and the biochemistry of life. The discovery of photoacids and photobases, i.e., molecules whose p*K*_a_ values decrease or increase in the electronically excited‐state,^[^ ^1^, ^2^ ^]^ opened up a variety of industrial and therapeutical applications of light‐driven intermolecular proton transfer. Using light as a tool, it is now possible to spatially and temporally manipulate proton transfer reactions, e.g., in the semiconductor industry, where photoacids and ‐bases are widely used for polymeric resists.^[^ ^3^, ^4^ ^]^ Lewis‐acidic catalysts for the Mukaiyama aldol reaction were generated by UV irradiation,^[^ ^5^ ^]^ photocurrents have been generated in electrochemical cells by the combined use of photoacids and photobases^[^ ^6^ ^]^ and it has recently been shown that seawater can be decarbonized with the help of photoacids.^[^ ^7^ ^]^

Common to all these approaches is the excitation of an organic photoacid or photobase with UV light. In an attempt to shift the wavelength for light‐controlled intermolecular proton transfer reactions to the visible region, photobase‐generators have been introduced that can be excited at wavelengths up to 660 nm.^[^ ^8^ ^]^ Unfortunately, such photobase generators are “single‐use molecules” and cannot undergo a second reaction cycle.

An alternative approach is to couple photobasic organic motifs to photoactive metal complexes, in which metal‐to‐ligand charge transfer (MLCT) transitions are available in the visible spectral range. In such a scenario, visible‐light excitation of the MLCT transition increases the electron density in the ligand, which carries the photobasic motif and, thus, renders it more prone to accept a proton. Hence, the excited‐state p*K*_a_* value increases compared to the ground‐state p*K*_a_. If a proton donor of lower or at least comparable p*K*_a_ is present in solution, optical excitation of the photobase is followed by excited‐state proton transfer (ESPT) from the acid to the photobase in its electronically excited‐state. If the complex acting as photobase is photostable, both as base and in its protonated conjugated acid form, the ground‐state base can be regenerated, a concept pioneered by Taube in 1968^[^ ^9^ ^]^ and later further explored by Peterson and Demas in 1976.^[^ ^10^ ^]^ In the latter case they utilized a variant of the archetypal [Ru(bpy)_3_]^2+^ complex, in which one of the bpy ligands is replaced by two cyanate ones (bpy: 2,2′‐bipyridine). While only the deprotonated form is emissive, the authors were able to study the excited singly and doubly protonated forms as a function of H^+^ concentration in aqueous solution. Soon after this seminal report, other Ru(II)‐containing systems which served as photoacids and photobases were reported^[^ ^11^, ^12^, ^13^, ^14^, ^15^, ^16^ ^]^ but also complexes based on different metal centers such as Re(I),^[^ ^16^, ^17^ ^]^ Os(II),^[^ ^18^, ^19^ ^]^ Pt(II),^[^ ^20^ ^]^ or Fe(II)^[^ ^21^ ^]^ have been explored in this context.^[^ ^22^ ^]^

Nonetheless, Ru(II)‐complexes are particularly well‐suited as photoacids and photobases as their extended ^3^MLCT lifetime provides a significant temporal window for intermolecular ESPT to occur. One approach that resulted in an exceptionally high ligand‐field splitting and, consequently, an increased lifetime was the use of 2,6‐di(quinolin‐8‐yl)pyridine (dqp), as a tridentate ligand.^[^ ^23^, ^24^ ^]^ Due to the increased bite angle of this ligand, which amounts to 179.6°, an almost perfect octahedral geometry was achieved in the corresponding [Ru(dqp)_2_]^2+^ complex. This resulted in a phosphorescence lifetime of 3 µs. Subsequently, the selective substitution of individual carbon atoms in the quinoline ring led to a marked modulation of the oxidation potential of the complex depending on the position of the introduced nitrogen.^[^ ^25^ ^]^

**Figure 1:**
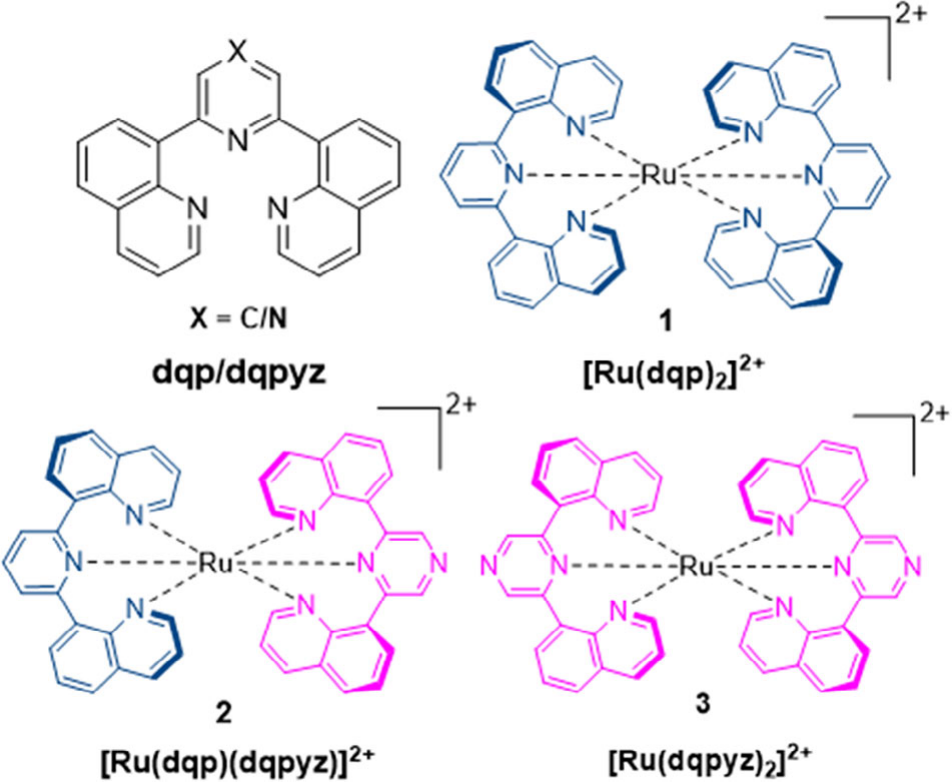
Schematic representation of the ligands 2,6‐di(quinoline‐8‐yl)pyridine/pyrazine (dqp/dqpyz) and their homoleptic and heteroleptic Ru(II) complexes (**1**/**3** and **2**, respectively.) In all cases, PF_6_^−^ is employed as counterion.

In this contribution, we present a novel version of the prototypical dqp ligand, in which the 4‐carbon atom in the central pyridine ring is substituted for nitrogen, yielding 2,6‐di(quinolin‐8‐yl)pyrazine (dqpyz). This pyrazine unit introduces a photobasic moiety in direct proximity to the metal center. At the same time, the nearly octahedral geometry is retained and this is what distinguishes our approach from other Ru(II)‐complexes with pyrazine‐containing ligands.^[^ ^9^, ^26^ ^]^ With both dqp and dqpyz at hand, this contribution reports the synthesis of both the homoleptic complexes [Ru(dqp)_2_]^2+^ and [Ru(dqpyz)_2_]^2+^ (**1** and **3**, respectively) as well as the heteroleptic counterpart [Ru(dqp)(dqpyz)]^2+^ (**2**) (Figure 1). Furthermore, the steady‐state and time‐resolved spectroscopic characterization of the complexes is reported. By varying the solvent and the pH value we obtain mechanistic insights into the intermolecular ESPT involving the complexes. The mechanistic studies combine pump‐wavelength‐dependent transient absorption (TA) and time‐resolved emission spectroscopy as well as (TD) DFT calculations.

## 2 Results and Discussion

### 2.1 Synthesis and Structural Characterization

In this study, we employed dqp [2,6‐di(quinolin‐8‐yl)pyridine] and dqpyz [2,6‐di(quinolin‐8‐yl)pyrazine] ligands to synthesize both homoleptic (**1** and **3**) and heteroleptic (**2**) Ru(II) metal complexes. The ligands were synthesized via Suzuki reaction, a well‐known cross‐coupling technique used to form carbon–carbon bonds between aryl halides and boronic acids, which allowed us to obtain the desired ligands with 55% yield. For the synthesis of the homoleptic metal complexes, we utilized a commercially available metal precursor, specifically Ru(DMSO)_4_Cl_2_, under appropriate conditions (see Supporting Information) and obtained the desired products, where the metal center is coordinated with identical ligands (yield: 20% for **1** and 22% for **3**).

The synthesis of the heteroleptic complex **2** required a more nuanced approach. First, we employed a Ru(II)‐based precursor [Ru(dqp)(NCMe)_3_]^2+^, which provides a well‐defined coordination environment, thus facilitating the formation of heteroleptic complexes. For this purpose, [Ru(dqp)(NCMe)_3_]^2+^ was synthesized following a published procedure.^[^ ^22^ ^]^ This precursor was then brought to reaction with the dqpyz ligand in refluxing acetonitrile overnight. Following the synthesis, the metal‐complexes were subjected to purification through column chromatography. This technique allowed the separation of the desired metal complexes from any unreacted starting materials and by‐products with an overall yield of 18%. After purification, a counterion exchange from Cl^−^ to PF_6_^−^ was performed with potassium hexafluorophosphate (KPF_6_), which increased the solubility of **2** in polar solvents.

**Figure 2:**
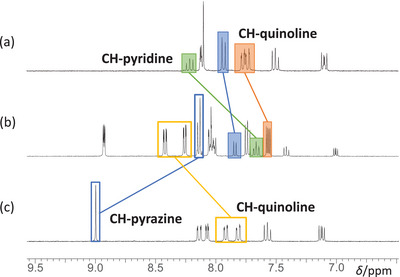
^1^H NMR‐spectra (CD_3_CN, 300 MHz, 25°C) of (a) mer‐[Ru(dqp)_2_]^2+^ (b) trans fac‐[Ru(dqp)(dqpyz)]^2+^ and (c) mer‐[Ru(dqpyz)_2_]^2+^ complexes. For clarity, only significant protons have been highlighted.

Analyzing the ^1^H NMR spectra in Figure 2 reveals distinct peak structures for each compound. The profound effects of ligand structure and metal complexation on the chemical shifts are highlighted. The homoleptic [Ru(dqpyz)_2_]^2+^ complex exhibits a notable downfield shift of the pyrazine proton to approximately 9.0 ppm, a clear indicator of metal coordination. This shift is attributed to the electron‐withdrawing nature of the pyrazine ring, further enhanced by ruthenium binding. The aromatic region (7.0–8.5 ppm) shows a complex pattern of multiplets, reflecting the symmetrical nature of the complex. In contrast, the free dqpyz ligand (see the Supporting Information) presents a simpler pattern with less peak splitting, particularly evident in the 7.5–8.5 ppm region. The absence of metal coordination allows for greater electron delocalization across the ligand, resulting in more shielded proton environments. The heteroleptic [Ru(dqp)(dqpyz)]^2+^ complex displays the most intricate pattern, a consequence of the asymmetry introduced by the two different ligands. The spectrum shows a broader distribution of peaks, with distinct signals for both pyridine and pyrazine protons. This complexity arises from the unique electronic environments created by the mixed ligand system, where each ligand experiences a slightly different influence from the metal center. The [Ru(dqp)_2_]^2+^ complex lacks the characteristic downfield pyrazine signal and exhibits a different distribution of peaks in the aromatic region compared to its pyrazine‐containing counterparts. The pyridine protons in this complex appear more shielded compared to the pyrazine analogues, reflecting the differing electronic properties of pyridine versus pyrazine. Across all complexes, metal coordination induces significant changes in the electronic environment of the ligands. This is evidenced by the altered multiplicity and chemical shifts of the quinoline protons, which appear as more resolved multiplets in the complexes compared to broader signals in the free ligand. These spectral differences underscore the substantial electronic modulation achieved through ligand variation and ruthenium coordination, pointing to potential differences in photophysical and electrochemical properties among these complexes.

**Figure 3:**
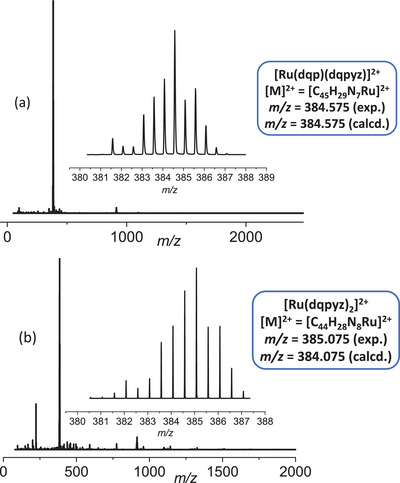
HR‐ESI‐TOF mass spectrum of (a) heteroleptic complex [Ru(dqp)(dqpyz)]^2+^ (**2**) and (b) homoleptic complex [Ru(dqpyz)_2_]^2+^ (**3**). The figure shows the comparison of the expected and calculated isotopic patterns of the respective [M]^2+^ peaks.

The ESI‐MS spectra in Figure 3 of both the heteroleptic and homoleptic complexes **2** and **3** display base peaks at *m*/*z* 384.575 and 385.075, respectively. These peaks correspond to the [Ru(L)_2_]^2+^ species, where L represents the ligand(s). For the heteroleptic complex (Figure 3a), the peak at *m*/*z* 384.575 results from both dqp and dqpyz; whereas, for the homoleptic complex (Figure 3b), the peak at *m*/*z* 385.075 results from its two dqpyz ligands. The slight difference in *m*/*z* values reflects the ligand variation, and both peaks indicate that the ruthenium exists predominantly in the +2‐oxidation state in the ionized form. The molecular structure of the complexes was then confirmed through single‐crystal XRD of their PF_6_^−^ salts.

### 2.2 X‐Ray Structure Analysis

**Figure 4:**
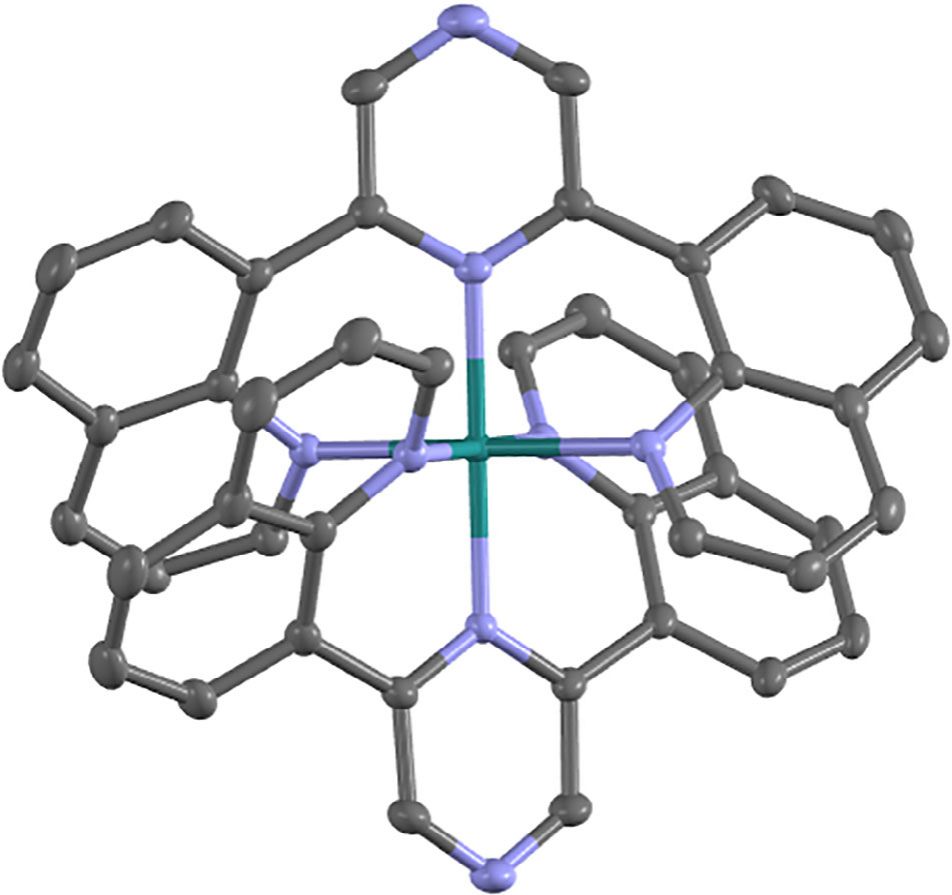
Solid‐state molecular structure of [Ru(dqpyz)_2_]^2+^ (**3**) as determined by single‐crystal XRD of [**3**](PF_6_)_2_ ·2 MeCN. Hydrogen atoms, counter ions and solvent molecules were omitted for clarity.

Figure 4 displays the X‐ray crystallographic data of complex **3**. In accordance with the NMR data, the two dqpyz ligands adopt a meridional coordination mode around the Ru(II) center. In comparison to typical [Ru(dqp)_2_]^2+^‐based complexes,^[^ ^27^ ^]^ complex **3** features a shorter Ru─N^pyz^ bond of 2.0163(16) Å, which indicates a stronger π‐back bonding (vide infra). The Ru─N^qu^ bond lengths as well as the internal N─Ru─N bond angles and the dihedral torsion with the tridentate ligand enforced by stacking agree well with those of the previously reported reference complex.

Next, the nuclear structure of the series of complexes and the respective protonated forms were analyzed by DFT (see the Supporting Information for details). As previously detailed and explained for complex **1**,^[^ ^28^ ^]^ the calculated Ru─N bond lengths are systematically overestimated by ca. 0.03 Å descending from the employed DFT methodology, while all internal N─Ru─N bond angles and the dihedral distortion within the ligand are reproduced well. More importantly, the replacement of one pyridine unit (**1**) to pyrazine (**2**) leads to a significantly shorter Ru─N^pyz^ bond length and the concomitant elongation of the remaining opposite Ru─N^py^ (Δ_Ru─N_ = 0.024 Å), which is well explained by the *trans*‐effect in coordination compounds. The protonation of the peripheral pyrazine N atom (**2H^+^**) enhances the *trans*‐effect and, thus, leads to significantly shorter Ru─N^pyzH+^ bond lengths versus Ru─N^py^ (Δ_Ru─N_ = 0.068 Å). The corresponding changes are also found for **3** and **3H^+^**, i.e., the replacement of the remaining dqp by a second dqpyz ligand partly compensates the *trans*‐effect in the singly protonated form (Δ_Ru─N_ = 0.047 Å). In all cases, the calculated structural changes in the N^−^Ru─N bond angles and the dihedral distortion are inferior. Selected structural parameter are summarized in Table 1.

**Table 1:** Selected structural data from crystallographic measurements and DFT calculations.^[^ ^a^ ^]^

Complex	Ru−N^py(z)^	Ru−N^qu^	N^qu^−Ru−N^py(z)^	N^qu^−Ru−N^qu^	N^py(z)^−Ru−N^py(z)^′	Dihedral distortion^[c]^
1 (X‐ray)^[b]^	2.031 (3)	2.074 (5)	89.6 (7)	178.9 (5)	178.7	38.1 ± 1.8
1 (DFT)	2.059	2.101	89.5	179.0	180	38.6
**2** (DFT)	2.062 2.038 (pyz)	2.103 2.105 (pyz)	89.4 89.4 (pyz)	178.7 178.7 (pyz)	180	38.7 37.7 (pyz)
**2**H^+^ (DFT)	2.070 2.002 (pyzH)	2.110 2.108 (pyzH)	88.7 89.7 (pyzH)	177.4 179.4 (pyz)	180	38.9 37.7 (pyz)
**3** (X‐ray)	2.016	2.071 (5)	90.1 (3)	179.7 (2)	179.9	35.9 ± 2.9
**3** (DFT)	2.042	2.107	89.2	178.4	180.0	37.8
**3**H^+^ (DFT)	2.055 2.008 (pyzH)	2.113 2.109 (pyzH)	88.5 89.6 (pyzH)	177.1 179.2 (pyzH)	180	38.1 37.7 (pyzH)
3H_2_^2+^ (DFT)	2.003	2.114	89.0	178.0	180	37.8

^[a]^ Numbers represent the average values and the difference to the maximal/minimal observed values. Bond lengths in Å and angles in deg.

^[b]^ Data for complex **1** taken from reference [27].

^[c]^ Between pyridine and quinoline unit along the interannular C─C bond.

### 2.3 Steady‐State and Transient Emission Properties of the Unprotonated Forms in CH_3_CN

**Figure 5:**
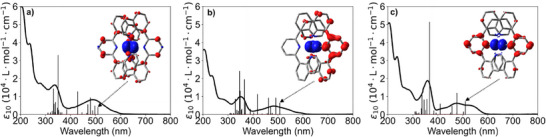
Extinction coefficients of the *mer*‐configurations of complexes **1**–**3** in CH_3_CN sorted in order a–c. The 40 calculated lowest‐energy transition energies and their corresponding oscillator strengths are displayed as vertical lines (scaled for better visibility). In the insets the electron density distribution following the lowest (2nd lowest for complex **2**) energy ^1^GS→^1^MLCT transition is shown with the isovalue drawn at 0.002. Blue and red represent lower and higher electron densities as compared to the ground state, respectively.

Figure 5 depicts the absorption spectra of complexes **1**–**3** taken in acetonitrile. All compounds show ligand‐centered π→π* transitions below 400 nm. The position of the dominant absorption peak in the near UV, however, depends on the specific compound and is observed in the range between 335 and 360 nm when the dqp ligand is substituted for dqpyz. We therefore attribute this absorption to be mainly pyridine/pyrazine centered. This is further supported by the fact, that free pyrazine in basic aqueous solutions absorbs at 374 nm.^[^ ^29^ ^]^ In the visible region, a broad MLCT absorption is visible between 400 and 600 nm for all compounds. This band broadens at the red flank when dqp is substituted for dqpyz.

The UV/Vis spectra are well explained by the results of TD‐DFT data. The corresponding electronic transitions reproduce the experimental bathochromic shifts as well as relative contributions to the ^1^MLCT envelope, including the known displacement towards higher energies (see oscillator strengths in Figure 5).^[^ ^28^ ^]^ The low‐energy transitions, which correspond to the long‐wavelength shoulder of the ^1^MLCT envelope, display a distinct charge transfer from Ru‐d orbitals to the pyrazine unit, in line with the localization of the involved frontier orbitals. The associated changes in electron density distribution are represented in the inset of Figure 5 with blue and red representing a lower or higher electron density, as compared to the ground state (^1^GS), respectively.

**Figure 6:**
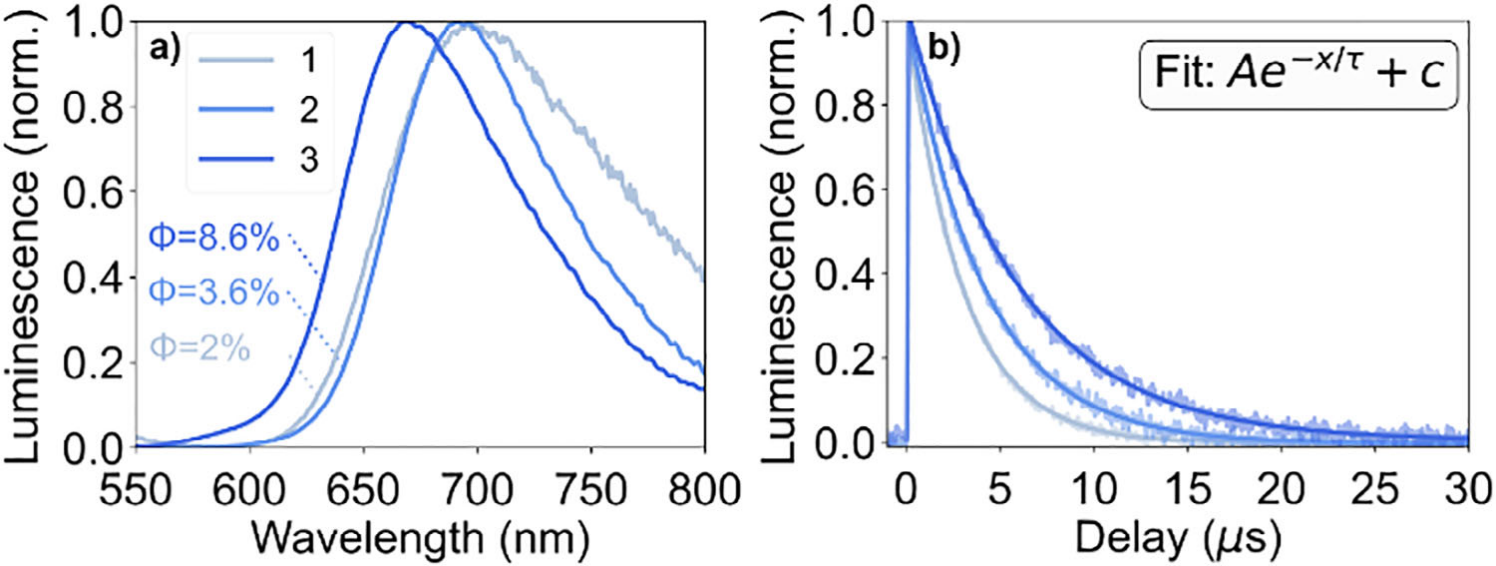
a) Normalized MLCT emission spectra of complexes 1–3 in inert CH_3_CN (after excitation at 350 nm) along with the respective quantum yields and b) emission traces (excited at 355 nm). Time‐resolved emission data were collected at the respective emission maximum and are displayed along with a monoexponential fit.

All compounds (in the *mer*‐conformation) are emissive when dissolved in CH_3_CN (see Figure 6a). The substitution of dqp for dqpyz leads to a blue‐shift of the emission from 698 to 670 nm reflecting an increase of the HOMO‐LUMO gap by 600 cm^−1^, which goes hand‐in‐hand with an increase in emission quantum yield from 2%^[^ ^23^ ^]^ to 8.6% (and a prolonged phosphorescence lifetime, which increases from 2.9 to 5.8 µs; see Table 2).^[^ ^30^ ^]^ Consistent with previous investigations of complex **1**,^[^ ^23^ ^]^ all complexes show a single‐exponential emission decay.

**Table 2:** Steady‐state and transient emission properties of compounds **1**–**3** in inert CH_3_CN at room temperature as well as basic and photobasic properties in aqueous media.

Compound	$\lambda_{\mathrm{em}}\;\left(\mathrm{nm}\right)$ ^[a]^	$\Phi_{\mathrm{ph}}\left(\%\right)$ ^[b]^	τ(μs) ^[b]^	p*K*_a_	p*K*_a_*^[c]^
**1** ^[d]^	698	2	2.9	–	–
**2**	692	3.6	4.1	−0.38 ± 0.05	8.8± 0.5
**3**	670	8.6	5.8	−0.54 ± 0.17	8.6 ± 0.5

Emission data collected after excitation at

^[a]^350 and

^[b]^355 nm.

^[c]^Determined from fluorescence titration experiments.

^[d]^Quantum yield value taken from Abrahamson et al.^[^ ^23^ ^]^ (measured in oxygen‐free MeOH/EtOH mixtures). Degradation of compound **1** is seen for aqueous solutions of pH < 0.

Furthermore, nanosecond TA experiments were conducted for all complexes (Figure S11). The data reveal a single‐exponential ground‐state recovery for all complexes, the spectral characteristics of which confirm that the long‐lived state is of ^3^MLCT character.^[^ ^23^ ^]^ The ns‐TA lifetimes, 2.5, 3.6 and 5.6 µs for **1, 2** and **3**, respectively, resemble the emission lifetimes corroborating that the long‐lived state is indeed an emissive ^3^MLCT excited‐state.

### 2.4 Determination of p*K*_a_ and p*K*_a_* in Aqueous Solutions

**Figure 7:**
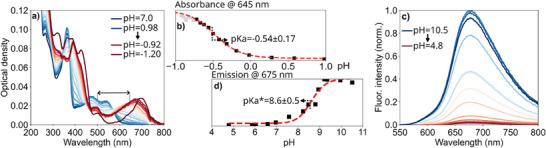
a) Spectrophotometric titration of complex **3** with H_2_SO_4_ in aqueous solution. b) Cross section of the data in a) at 645 nm and logistic fit for pH values above −0.75. The function $\frac{a\;-\;b}{1+{\left(x/c\right)}^{d}}+b$ was used for the fit and the whole data set was shifted by 5 pH units during this process. The obtained p*K*_a_ and respective error are averaged over the wavelength range 600–700 nm. The theoretical pH‐values are calculated assuming that H_2_SO_4_ dissociates one of its hydrogen atoms and the absolute absorbance values are concentration corrected. c) Fluorescence spectra in different pH buffer solutions after excitation at 350 nm. d) Fluorescence intensities at 675 nm with the same logistic fit used in b). The equivalence point is calculated as the average between 600 and 750 nm and the error is given by the accuracy of the used pH‐meter.

We assess the ground‐ and excited‐state basicity of compounds **2** and **3**, based on absorption and fluorescence titration experiments. Due to its longer excited‐state lifetime (and therefore higher probability of diffusion controlled photobasic reactions) and due to the presence of two possible protonation sites, we limit our discussion here to complex **3**. A summary of all the relevant data is shown in Figure 7. For the initial part of the titration, i.e., for the first protonation step, very similar results are obtained for complexes **2** and **3** and the corresponding analysis for **2** is therefore shifted to the supporting information (see Figure S15).

Figure 7a depicts the absorption spectra of **3** in aqueous media upon adding H_2_SO_4_. The spectra recorded at pH 7.0 and pH 0.98 are essentially identical, ruling out any spectral contamination (e.g., from degraded complex). However, at pH −0.75 both the ligand‐centered and MLCT absorption bands shift to longer wavelengths as a result of protonation. It is noteworthy that the MLCT band shifts even further to the red when the pH value is reduced to −0.92, an effect that becomes even clearer with a separate measurement at pH −1.20. As similar spectral shifts at very low pH values are not observed for the heteroleptic complex **2**, we associate this behavior with a sequential protonation of both dqpyz ligands in **3,** yielding stepwise **3H^+^** and **3H_2_^2+^**. The distinct pH values at which the two protonations occur indicate an electronic interaction of the two photobasic sites associated with the two dqpyz ligands. The p*K*_a_ of the first protonation is estimated to −0.54 ± 0.17 based on the absorption data in the pH range between 0.98 and −0.75 (see Figure 7b). Notably, a similar value, i.e., −0.38 ± 0.05, is obtained for the p*K*_a_ of the dqpyz ligand in **2**. This slightly increased value is in line with the stronger electron‐withdrawing effect of the pyrazine unit in the second ligand of **3**, as compared to pyridine in **2**.

To probe the excited‐state basicity of the investigated compounds, pH‐dependent emission studies were performed for all complexes. Experiments at pH 7 and pH 1 (Figure S12) show differences in the emission intensities of ligand and MLCT bands for complex **1**. Upon decreasing the pH value by adding H_2_SO_4_ the ligand emission increases, while the MLCT emission decreases. Since protonation is not possible for complex **1**, these changes in the Franck–Condon factors must be associated with the different solvation environments. The absence of unbound ligands, which might also result in an increased ligand emission, is further confirmed by absorption measurements at pH 7 and pH 1 (see Figure S13). This method presents a simple test for the possible protonation of the unbound quinoline moiety with a p*K*_a_ of ca. 5.^[^ ^31^ ^]^ For **2** and **3**, similar features are observed for the ligand‐based emissions, i.e., upon acidifying the solvent the emission increases but for the ^3^MLCT emission, on the other hand, no emission is observed at all. These results already indicate that ESPT might lead to a nonradiative decay process.

The absence of emission from (the presumed) **2H^+^** and **3H^+^** makes it impossible to estimate the p*K*_a_* values of **2** and **3** via Förster analysis.^[^ ^2^, ^32^ ^]^ Therefore, we approximate the energy of the $\upsilon_{0\to 0}$ transition based on the absorption spectra only.^[^ ^33^ ^]^ A graphical representation of the involved thermodynamic cycle and formulas can be found in Figure S14.^[^ ^32^ ^]^ Considering the energetic difference of 4375 cm^−1^ between the MLCT bands of unprotonated and singly protonated species (indicated by the horizontal arrow in Figure 7a), we estimate the p*K*_a_* value of **3** to be 8.7. Compared to the ground‐state, this amounts to an increase in basicity in the excited‐state (Δp*K*_a_) of 9.2. To corroborate this estimate, phosphorescence titration experiments in different pH‐buffered solutions were performed (Figure 7c). As ^3^MLCT emission is only possible for pH values above the p*K*_a_*, while at lower pH values the nonradiative decay channel dominates, we can use the pH dependence of the emission intensity to estimate the p*K*_a_* value. Using a logistic function to fit the pH dependence of the emission intensity yields p*K*_a_* = 8.6 ± 0.5, a value that is in line with the estimate of the excited‐state basicity based on the absorption spectra (Figure 7d). A very similar value of 8.8 ± 0.5 is obtained for the heteroleptic complex **2**. Taking the slightly higher ground‐state p*K*_a_ of **2** into account, we conclude that the increase in basicity is almost the same for **2** and **3**, indicating that the photobasic excited‐state is not dependent on the electron withdrawing effect of the second ligand.

### 2.5 TD‐DFT Calculations of 3, 3H^+^ and 3H_2_^2+^

**Figure 8:**
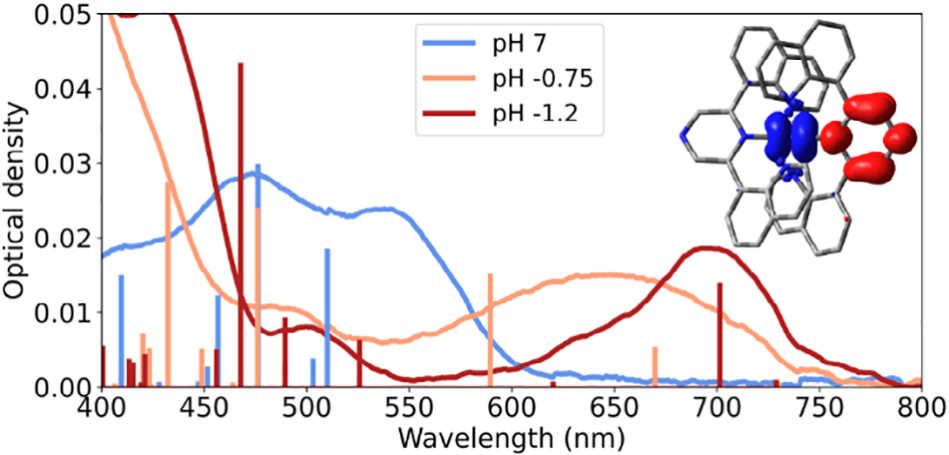
Experimental spectra of complex **3** in aqueous solutions of different acidity. Depending on the pH, the spectra are assigned to either **3**, **3H** ^**+**^ or **3H**_**2**_^**2+**^. The calculated transitions for the different species are shown as vertical lines. The inset shows an electron‐density difference map for the lowest energy transition of **3H** ^**+**^. Isovalue drawn at 0.002.

Our spectroscopic assignment is supported by TD‐DFT calculations. Figure 8 displays the absorption spectra recorded at pH 7, −0.75 and −1.2 alongside calculated transition energies of **3**, **3H^+^** and **3H_2_^2+^**. Notably, at pH −1.2 some **3H** ^+^ likely remains unprotonated, but the MLCT absorption of **3H_2_^2+^** is already clearly visible and is in perfect agreement with the calculated transition energy. Good agreement between theory and experiment is also observed for the unprotonated and singly protonated species. The inset of Figure 8 shows an electron density difference map between the ground‐ and lowest‐excited state of **3H^+^**, highlighting the strongly directional nature of the MLCT transition as electron density is displaced from the metal center to the protonated pyrazine unit. This is in contrast to the unprotonated species **3** (see Figure 5c) where the ^1^MLCT state is delocalized over both pyrazine moieties.

### 2.6 Photoinduced ESPT Dynamics

**Figure 9:**
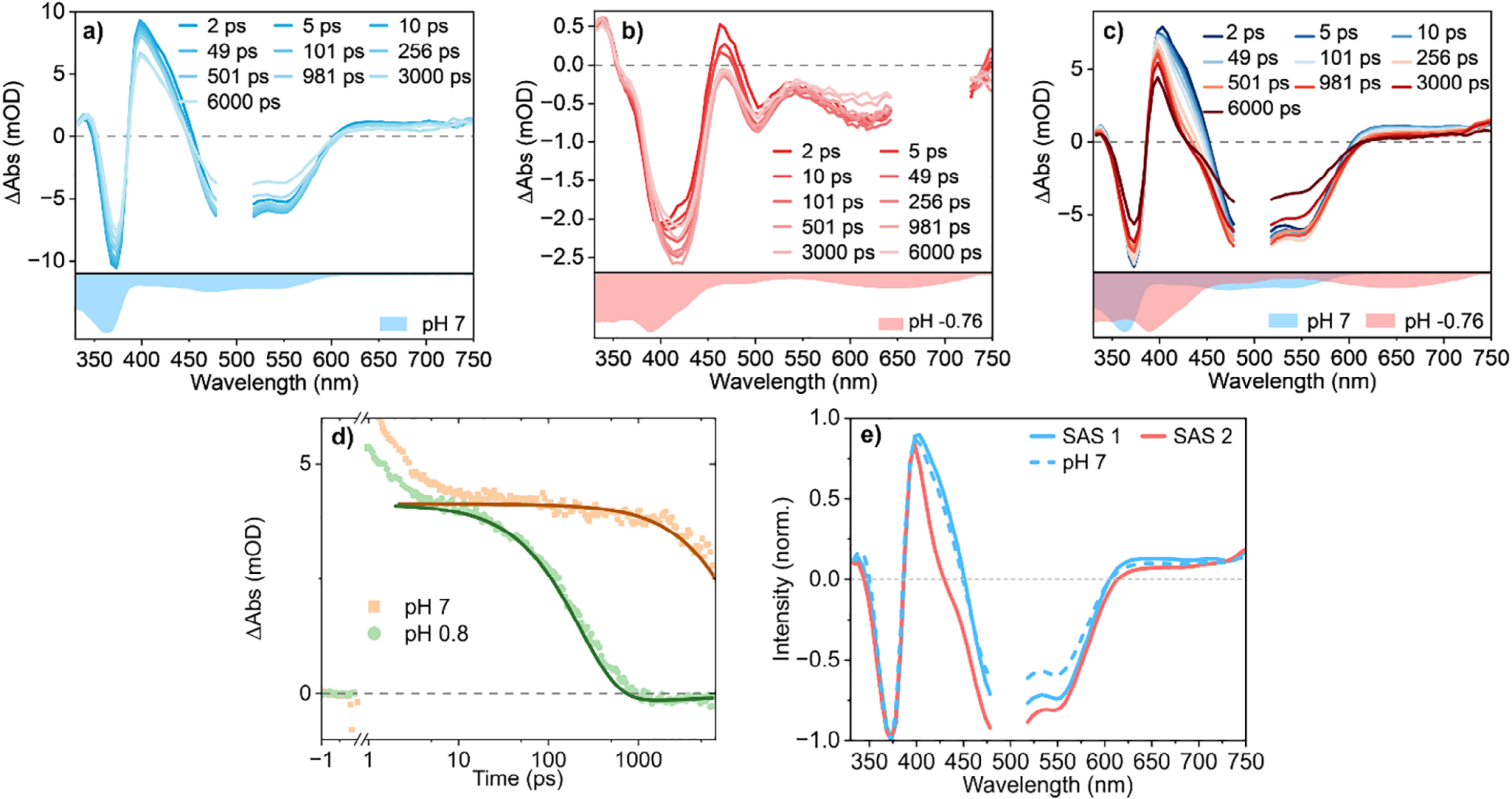
Transient absorption data of complex **3** in aqueous solutions of a) pH 7, b) pH −0.75 and c) pH 0.8 acidified with H_2_SO_4_. In a) and c) the ground‐state deprotonated forms were each excited at 490 nm while the MLCT state of the protonated form in b) was excited with pulses centered around 680 nm. d) Elucidates the differences in the kinetics at pH 7 and 0.8 due to ESPT at 430 nm. In all cases only values above 2 ps were considered for the global fit, which yields the (normalized) SAS in e) for pH 0.8 on which the SAS of the measurement at pH 7 is superimposed.

In order to resolve the kinetics of the ESPT reaction between the complex and the solvent, TA experiments were performed in aqueous solutions of varying pH value. Solutions with pH 7, 0.8 and −0.75 were prepared by adding various amounts of H_2_SO_4_ to the aqueous solution. As a consequence, **3** will be studied both in the ground‐state unprotonated form (pH 7 and 0.8) or in the singly protonated state **3H** ^+^ (pH −0.75). Figure 9 shows the TA spectra of **3/3H** ^+^ at the different pH values, recorded upon MLCT excitation at 490 or 680 nm. The TA spectra obtained for the unprotonated form (pH 7, Figure 9a) display strong ground‐state bleach (GSB) between 350 to 385 nm and 450 to 600 nm. These features line up with the ligand‐based and MLCT transitions dominating the steady‐state absorption spectrum of the complex shown in the bottom part of the figure for comparison. Similarly, the ground‐state protonated complex (pH −0.75, Figure 9b) shows prominent GSB features, which—due to protonation—appear red‐shifted between 350 to 450 nm and 480 to 750 nm. For both protonation states exited‐state absorption (ESA) features are visible between the GSBs and for very short probe wavelengths below ca. 350 nm. At long probe‐wavelengths bleach features dominate the spectrum of the singly protonated complex, while a flat, unstructured ESA is apparent at pH 7, which resembles the ligand‐to‐metal charge‐transfer transitions typically observed in Ru(II)‐polypyridyl complexes in this spectral range.^[^ ^24^, ^34^, ^35^, ^36^ ^]^

As time evolves, the TA signatures of the unprotonated species gradually decrease within the experimental window of 7 ns and across the probe spectrum. In the protonated case, an initial rise in the ligand GSB feature is observed on a time scale of approximately 100 ps, which is then followed by a gradual decay.

Global analysis^[^ ^37^ ^]^ indicates that the TA data recorded at pH 7 can be accounted for by a single exponential decay (see Table 3); whereas, a bi‐exponential fit is necessary to account for the data of the protonated form (pH −0.75). Absorption and emission spectroscopy at pH −0.75 revealed that the complex remains in its protonated form both in the electronic ground and excited state. Hence, we conclude that the additional kinetic component, *τ* = 44 ps, is not due to changes of the protonation state of the complex. We rather hypothesize that it is associated with solvent reorganization. The comparably slow solvent reorganization is due to the high concentration of H^+^ (5.6 M) and HSO₄⁻ ions in solution. The solvent molecules in the solvation shells of the ions respond less rapid to electronic changes in the solute.^[^ ^38^ ^]^ In contrast, at pH 0.8 the solution contains only 0.2 M of dissociated ions and the solution at pH 7 comprises only H^+^ ions that result from water's autoprotolysis. In the latter case, solvent reorganization effects are well studied and known to occur within hundreds of fs after optical excitation.^[^ ^39^, ^40^, ^41^ ^]^ We also acknowledge the intrinsic concentration of PF_6_⁻counter anions in solution. However, given that only µM concentrations of complex **3** are used in these experiments, the effect of these ions on the photochemistry should be marginal. In order to focus on the ESPT reactivity of the complex‐photobase rather than solvation effects, we restrict the further discussion to TA data recorded at delay times >2 ps and assume that, at both pH 7 and 0.8, solvent reorganization is finished at this time. At the same time, ^1^MLCT→^3^MLCT intersystem crossing (ISC) will be completed^[^ ^42^ ^]^ and, hence, we start the discussion from the assumption that the excited‐state captured in the TA signal at 2 ps is of ^3^MLCT character.

**Table 3:** Fitted time constants for complexes 1–3 in non‐inert aqueous solutions following MLCT excitation.

Compound/solvent	$\lambda_{\mathrm{exc}}\left(\mathrm{nm}\right)$	$\tau_{\mathrm{unprot}}\;\left(\mathrm{ns}\right)$ ^[a]^	$\tau_{\mathrm{ESPT}}\;\left(\mathrm{ps}\right)$	$\tau_{\mathrm{prot}}\;\left(\mathrm{ns}\right)$ ^[a]^
**1**/pH 7	490	12 ± 2		
**1**/pH 0.8	490	15		
**2**/pH 7	490	13 ± 1		
**2**/pH 0.8	490	long^[b]^	351	long^[b]^
**3**/pH 7	490	15 ± 1		
**3**/pH 0.8	490	long^[b]^	243 ± 3	long^[b]^
Compound/solvent	$\lambda_{\mathrm{exc}}\left(\mathrm{nm}\right)$	$\tau_{\mathrm{sol}}\;\left(\mathrm{ps}\right)$	$\tau_{\mathrm{prot}}\;\left(\mathrm{ns}\right)$	
**3**/pH −0.75	680	44 ± 3	31 ± 1	

^[a]^ The presented measurement errors correspond to the standard deviations obtained from separate measurements.

^[b]^ Due to the nature of the applied fitting procedure, an accurate determination of the lifetimes of the unprotonated and protonated forms is not possible in this case. Depending on the selected start values, fit constants on the order of 10–30 ns are obtained for the lifetimes of the unprotonated and protonated forms.

From this point onwards, different photophysics are observed for the unprotonated and protonated forms, respectively. While the unprotonated species undergoes radiative relaxation back to the ground‐state, a nonradiative decay pathway is observed for the protonated one. Given the pH values of the solutions, we want to stress that the aforementioned molecular reaction pathways are not connected via ESPT. To target the kinetics of ESPT, we shift the focus to the measurement at pH 0.8 (see Figure 9c), at which we expect that the complex will be unprotonated in the electronic ground‐state but will act as a base in the electronically excited‐state. The TA spectra at pH 0.8 recorded at a delay time of 2 ps show GSB and ESA signatures similar to those observed at pH 7. However, upon increasing the delay times (10–1000 ps) notable kinetic differences become apparent: These differences are particularly evident when considering the narrowing of the ESA band at 430 nm (see Figure 9c,d). Figure 9d compares these asymmetries in the TA kinetics recorded at 430 nm at pH 7 and 0.8. While for pH 7 a gradual decay of the ESA band is only visible from 1 ns onwards, the transient signals for pH 0.8 already decrease in the range of 10–1000 ps.

**Figure 10:**
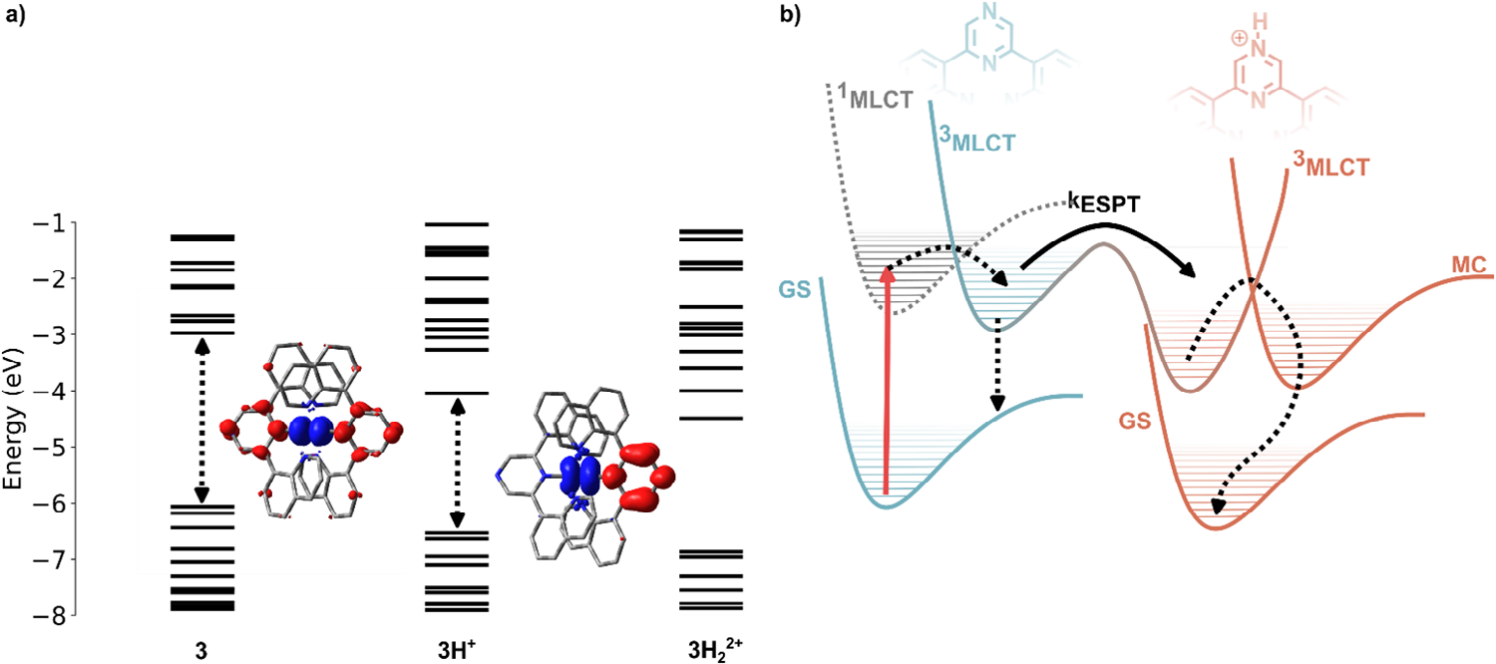
a) Calculated energy levels for **3**, **3H** **^+^** and **3H**_**2**_^**2+**^. For the former two the lowest energy ^1^GS→^1^MLCT transitions are depicted with dotted arrows and the resulting redistributions of electron density in the excited state are shown alongside. Isovalues were drawn at 0.002. b) Proposed ESPT mechanism between **3** and **3H** ^+^ along with the involved electronic states.

To explain the differences in the TA signals and to relate them to ESPT, which is expected to take place at pH 0.8, the model presented in Figure 10b is fitted globally. The model assumes a ^3^MLCT state as starting point for the fit. From this state, the system can either relax back to the ground‐state (dotted arrow) or undergo the ESPT pathway (solid arrow) followed by nonradiative decay (dotted arrow) to the ground‐state of the protonated form. The energetically downhill nature of the latter pathway agrees with the orbital energies calculated via TD‐DFT in Figure 10a and the absence of emission from **3H** ^+^ is in line with previous steady‐state emission measurements at pH 1 (see Figure S12). We propose that this nonradiative pathway takes place via metal‐centered (MC) states which elude detection in the TA data due to their short lifetime. Thus, fitting of the data collected at pH 0.8 with this model yields two species‐associated spectra (SAS), which are presented in Figure 9e. It is striking that the earlier one (SAS1) closely resembles the SAS obtained from the measurement at pH 7. This underlines that the complex is indeed unprotonated in the first ps following optical excitation. For the later times SAS2 is obtained which clearly reflects the narrowing of the ESA band at 430 nm (vide supra). Nevertheless, upon ESPT one might expect to see a clearer rise of the ESA signatures associated with the buildup of the protonated species along with a decay of the features associated with the unprotonated form. We ascribe the absence of such observables to a residual population of unprotonated complexes. This implicates that SAS2 does not only include the signatures of the protonated form but rather features contributions from the unprotonated form as well. Under the assumption that the extinction coefficients of both protonation states are similar (see Figure 7a), it is likely that the shift in the respective absorption bands results in some TA signatures canceling out. For example, the protonated form is characterized by weak ESA features centered around 470 nm which overlap with strong (MLCT) GSB features from the unprotonated form at the same wavelength. At the same time, the already existing ESA band from the unprotonated species, centered at 420 nm, is expected to decrease in intensity. The combination of these effects might explain the fact that the narrowing of the ESA band at 430 nm is not accompanied by a rise in the ESA bands characteristic to the protonated form. For the fraction of the excited‐state population that does undergo ESPT, kinetic modelling allows us to extract the proton transfer time (*τ_ESPT_*) of 243 ps for **3**. The same model was also applied to the heteroleptic complex **2**, yielding a somewhat longer proton transfer time of 351 ps (Figure S16). In this case a separate measurement of the protonated form was not possible as the complex degrades in acidic media of pH −0.75.

The proton transfer times obtained for **2** and **3** fall in the range of typical intermolecular ESPT processes, which proceed in the time range between some‐10 ps and ns; ESPT occurs within 23 ps in 5‐methoxyquinoline^[^ ^43^ ^]^ while ESPT in 6‐methoxyquinoline takes place in 1900 ps.^[^ ^44^ ^]^ Ultimately, the ESPT rate constants depend on several factors such as p*K*_a_*,^[^ ^43^ ^]^ temperature,^[^ ^45^, ^46^ ^]^ solvent composition,^[^ ^47^ ^]^ isotope effects^[^ ^48^, ^49^ ^]^ and salt concentrations.^[^ ^50^, ^51^, ^52^ ^]^

#### 2.6.1 Control Experiments—Complex **1**

For comparison, TA spectroscopy at pH 7 and pH 0.8 was also performed for complex **1** with excitation at 490 nm. Figure S17 reveals no significant differences between the two measurements, and, in both cases, the dynamics are governed by single exponential decays. A summary of all the time constants and fitting models can be found in Table 3.

#### 2.6.2 Control Experiments—Direct Excitation of the Pyrazine Unit

**Figure 11:**
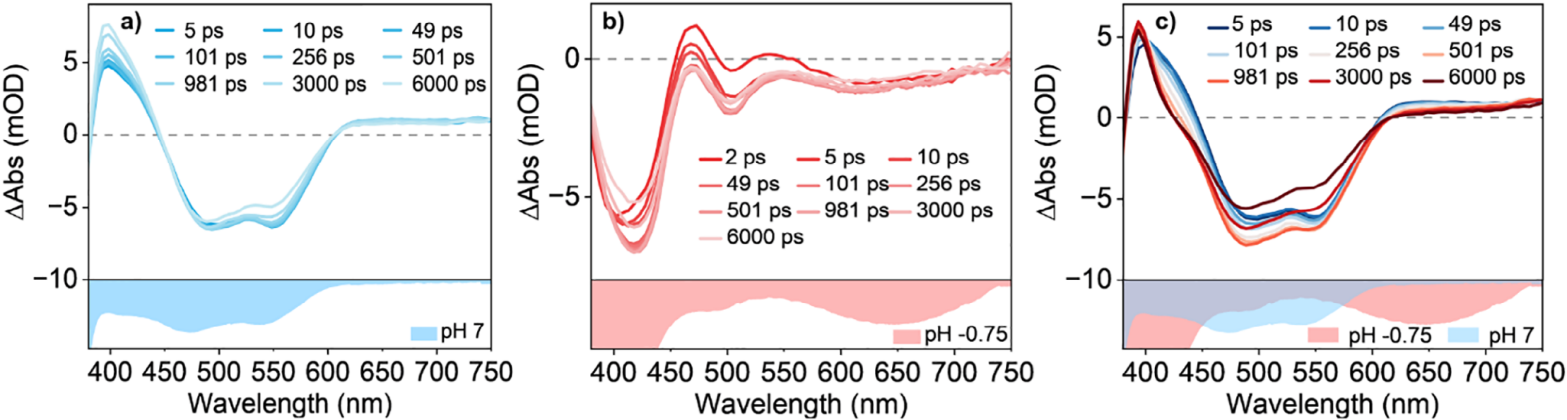
Transient absorption data of complex **3** in aqueous solutions of a) pH 7, b) pH −0.75 and c) pH 0.8 acidified with H_2_SO_4_ after ligand‐centered excitation (*λ*_exc_ = 350 nm). In all cases only values above 2 ps were considered for the global fits. The corresponding absorption spectra of the unprotonated and singly protonated species are shown in the bottom parts of every diagram.

To probe if the intrinsic photobasic properties of the pyrazine unit are preserved once it is coordinating the Ru(II) ion as part of the dqpyz ligand, we performed TA spectroscopy of **3** upon excitation at 350 nm. At this wavelength the absorption of the complex is dominated by ππ* absorptions of the pyrazine unit itself. This scenario leads to more complex TA features compared to direct MLCT excitation as seen in Figure 11. Although similar GSB signatures are observed between 450 to 600 nm for the unprotonated and between 380 to 450 and 480 to 750 nm for the protonated form, the observed ESA features are partially different. This is especially evident for the measurement at pH 7 in the wavelength range 380 to 450 nm, in which a gradual increase of the signal is observed until the end of the measurement window. Simultaneously, the low‐energy ESA band, characteristic of ^3^MLCT states, is still visible at wavelengths above 600 nm. Very similar results are also observed for complex **1** following excitation at 350 nm (see Figure S17). This leads us to presume, that another excited‐state, with possible ligand‐centered character, is also involved in these dynamics.

Similarly to the measurement at pH 7, the data at pH 0.8 also shows a small increase of the ESA band in the range between 380 and 420 nm, suggesting that this additional state also plays a role for complex **3**. Nevertheless, the narrowing of the ESA band in the range 420 to 450 nm, characteristic of ESPT, is still clearly visible and, therefore, leads us to conclude that proton transfer also plays a dominant role in the dynamics following ligand‐based excitation. Under the approximation, that this additional pathway does not play a predominant role in the observed photophysics, we employ the same model as shown in Figure 10b and arrive at a ESPT time constant of 337 ps. Finally, we want to point out that the dynamics of the singly protonated species are also slightly different with the solvent reorganization now being completed in 16 instead of 44 ps. A summary of all the fit constants obtained for complex **3** with excitation at 350 nm can be found in Table 4.

**Table 4:** Fitted time constants for complex **3** in non‐inert aqueous solutions following ligand‐centered excitation.

Compound/ solvent	$\lambda_{\mathrm{exc}}\left(\mathrm{nm}\right)$	$\tau_{\mathrm{unprot}}\;\left(\mathrm{ns}\right)$	$\tau_{\mathrm{unprot}2}\;\left(\mathrm{ns}\right)$	
**3**/pH 7	350	5 ± 2	18 ± 8	
Compound/solvent	$\lambda_{\mathrm{exc}}\left(\mathrm{nm}\right)$	$\tau_{\mathrm{unprot}}\;\left(\mathrm{ns}\right)$	$\tau_{\mathrm{ESPT}}\;\left(\mathrm{ps}\right)$	$\tau_{\mathrm{prot}}\;\left(\mathrm{ns}\right)$
**3**/pH 0.8^[a]^	350	long	337 ± 13	long
Compound/solvent	$\lambda_{\mathrm{exc}}\left(\mathrm{nm}\right)$	$\tau_{\mathrm{sol}}\;\left(\mathrm{ps}\right)$	$\tau_{\mathrm{prot}}\;\left(\mathrm{ns}\right)$	
**3**/pH −0.75	350	16 ± 1	22 ± 1	

*Note*: The presented measurement errors correspond to the standard deviations obtained from separate measurements.

^[a]^ Depending on the selected start values, fit constants on the order of 15–20 ns are obtained for the lifetimes of the unprotonated and protonated forms.

## 3 Conclusion

We report on the synthesis of two novel pyrazine‐containing Ru(II)‐complexes. These complexes are essentially isostructural to the well‐known Ru(II) bis‐dqp complex. Due to the ligand design, which yields almost perfect octahedral geometry of the resulting complexes, the MC states can be efficiently destabilized. On the other hand, we observe both an increase in quantum yield from 2% to 8.6% and emission lifetimes from 2.9 to 5.8 µs, for the pyrazine‐functionalized [Ru(dqpyz)_2_]^2+^ complex in comparison to the parent [Ru(dqp)_2_]^2+^ complex. Accompanied by a blue shift in the emission wavelength as dqp is substituted for dqpyz, we associate these changes to ^3^MLCT states which are progressively higher in energy as supported by DFT calculations.

In aqueous solutions we observe protonation of the pyrazine moieties at very low pH values upon addition of sufficient H_2_SO_4_. For both the heteroleptic [Ru(dqp)(dqpyz)]^2+^ and homoleptic [Ru(dqpyz)_2_]^2+^ complexes, similar p*K*_a_ values of −0.38 and −0.54 are obtained for the first protonation step. In addition, a second protonation is observed for [Ru(dqpyz)_2_]^2+^ at even lower pH values. This process is also accompanied by red shifts in the MLCT absorption bands as the dqpyz ligands become better π‐acceptors. In emission spectroscopy, the phosphorescent ^3^MLCT emissions are seen to disappear for both ground‐state and excited‐state protonation. This property can be used to determine the p*K*_a_* of the complexes, yielding values of 8.8 and 8.6 for [Ru(dqp)(dqpyz)]^2+^ and [Ru(dqpyz)_2_]^2+^, respectively. If protonation is not possible, as it is the case for [Ru(dqp)_2_]^2+^, this behavior is not observed.

The photobase design presented here significantly reduces the energy required for ESPT compared to free pyrazine in aqueous solution. fs‐TA spectroscopy revealed the ESPT dynamics both upon direct excitation of the pyrazine moiety and upon excitation of the MLCT transitions. We find that in the latter case, ESPT takes place from a ^3^MLCT state with a time constant of 243 ps for [Ru(dqpyz)_2_]^2+^. For the heteroleptic [Ru(dqp)(dqpyz)]^2+^ complex a similar time constant of 351 ps is obtained. In contrast, the excited‐state dynamics following direct excitation of the pyrazine moiety are more complicated and seem to involve a second ligand‐centered state. Nevertheless, ESPT is still observed with a time constant of 337 ps for [Ru(dqpyz)_2_]^2+^.

At last, we also tested the practical applicability of [Ru(dqpyz)_2_]^2+^ as a photobasic catalyst in the Henry‐type reaction of nitroethane and benzaldehyde. These preliminary experiments indicate that a reaction might be taking place but that its yield is not sufficient to unambiguously characterize the formed product (see Figures S18–S20 and Table S2). We speculate that this is due to the p*K*_a_ of nitroethane being on the same order of magnitude as the p*K*_a_* of [Ru(dqpyz)_2_]^2+^, i.e., 8.5^[^ ^53^ ^]^ and 8.6 ± 0.5, respectively. In the future, we plan to further optimize the ligand design to obtain photobasic moieties with p*K*_a_/p*K*_a_* values high enough to catalyze such reactions.

## 4 Experimental Section

### X‐ray crystallography

Single crystals of [Ru(dqpyz)_2_](PF_6_)_2_ (**3**) ∙2 CH_3_CN were obtained by vapor diffusion of diethyl ether into CH_3_CN solutions of the respective complex salts. The X‐ray diffraction intensity data were collected on a Bruker–Nonius Kappa‐CCD diffractometer equipped with a Mo‐Kα IµS microfocus source and an Apex2 CCD detector, at *T* = 120(2) K. The crystal structures were solved with SHELXT‐2018/3^[^ ^54^ ^]^ and refined by full matrix least‐squares methods on *F* ^2^ with SHELXL‐2018/3,^[^ ^55^ ^]^using the Olex2 1.5 environment.^[^ ^56^ ^]^ Multi‐scan absorption correction was applied to the intensity data.^[^ ^57^ ^]^ CCDC 2377177 contains the supplementary crystallographic data for this paper (see Table S1). These data can be obtained free of charge from The Cambridge Crystallographic Data Centre (CCDC; http://www.ccdc.cam.ac.uk).

### Sample preparation

For both steady‐state and time‐resolved experiments, spectroscopy grade solvents were purchased from commercial suppliers and used as received. All measurements were conducted in 10 mm quartz cells. Absorption measurements in CH_3_CN were performed in aerated solutions while emission, quantum yield and time‐resolved emission measurements were conducted in inert CH_3_CN solutions obtained via the freeze‐pump‐thaw method. All aqueous samples were prepared by mixture of deionized water, small amounts of highly concentrated CH_3_CN stock solutions and, if applicable, sulfuric acid.

### Steady‐state spectroscopy

Steady‐state absorption spectra were collected on a V‐670 UV/Vis/NIR spectrophotometer (JASCO, Germany). Steady state emission spectra were recorded on a FLS980 emission spectrometer (Edinburgh Instruments, UK) using a Xe lamp (ozone free 450 W) as excitation source. Fluorescence quantum yields were determined using the latter device in combination with an Ulbricht integrating sphere.

### ns‐Transient absorption measurements

Phosphorescence lifetimes and ns‐TA spectra were acquired using a custom‐built Nd:YAG laser setup with a 10 Hz repetition rate (Pascher Instruments, Sweden). Details regarding the experimental setup can be found elsewhere.^[^ ^58^ ^]^ All samples were excited with pulses centered around 355 nm, pulse energies of 700 µJ and optical densities of 0.2–0.5 at the excitation wavelength.

### fs‐Transient absorption measurements

Ultrafast TA spectra were collected using a custom‐built (Pascher Instruments, Sweden) setup with a 1 kHz Ti:Sapphire regenerative amplifier (Astrella, Coherant) as the laser source. The details of this setup are published elsewhere.^[^ ^59^ ^]^ Pump pulses centered around 350, 490, or 680 nm were generated in an optical parametric amplifier (TOPAS prime, Light conversion), while probe pulses were generated by a rotating CaF_2_ crystal.

All measurements were conducted with pump pulse energies between 500 and 700 nJ and optical densities of 0.2–0.7 at the excitation wavelengths. Absorption spectra were taken before and after each measurement to check for sample degradations.

Data analysis was carried out using the Kimopack Python Package.^[^ ^37^ ^]^ Prior to global fitting, all measurements were numerically corrected for the chirp of the white‐light probe and the data range −1 to 2 ps was excluded from the fit to avoid contributions from the coherent artifact.^[^ ^60^, ^61^ ^]^

### TD‐DFT calculations

The theoretical calculations based on density functional theory (DFT) were performed as reported previously.^[^ ^27^ ^]^ All calculations were performed with the Gaussian16 Program Package (Version A.02).^[^ ^62^ ^]^ The functional B3LYP^[^ ^63^, ^64^ ^]^ with empirical dispersion correction (GD3) was used, using the 6–31G* basis set for all atoms except Ru, which was described by an effective core potential and the associated orbitals (mwb28). For all calculation, the solvent environment was modelled for acetonitrile using the implemented polarization continuum model (PCM).^[^ ^65^, ^66^ ^]^ The corresponding geometries of the singlet ground states and triplet excited states were optimized from reasonable initial estimates. The true nature of all minima structures was confirmed by vibrational analysis showing no imaginary frequencies. The graphical visualizations of the three‐dimensional representations were generated by GaussView6.0.16.^[^ ^67^ ^]^ The vertical transitions are visualized by electron‐density difference maps (EDDMs), which depict the redistribution of electron density by regions of accumulation (red) and depletion (blue) of electron density using Gaussum3.0.^[^ ^68^ ^]^

## Conflict of Interests

The authors declare no conflicts of interest.

## Supporting information



Supporting Information

## Data Availability

The data that support the findings of this study are available from the corresponding author upon reasonable request.
