# Supplementary material for: Pyrazine‐Functionalized Ru(II)‐Complexes as Visible‐Light Activated Photobases
Source: Chemistry. 2025 Apr 28;31(36):e202404033. doi: 10.1002/chem.202404033 (PMC12202845; doi:10.1002/chem.202404033)
Supplement: Supplementary file 1 — Supporting Information [file CHEM-31-e202404033-s001.pdf]

Supporting information

Pyrazine-functionalized Ru(II)-complexes as visible-light activated photobases

Niklas Klosterhalfen[a,b] ‡, Nishi Singh[c,d] ‡, Michael Jäger[c,d], Andreas Winter[c,d], Phil Köhler[e], Ulrich S. Schubert*[c,d], Benjamin Dietzek-Ivanšić*[a,b]

[a] N. Klosterhalfen, Prof. Dr. B. Dietzek-Ivanšić
Department Functional Interfaces,
Leibniz Institute of Photonic Technology (Leibniz-IPHT)
Albert-Einstein-Str. 9, 07745 Jena, Germany
E-mail: benjamin.dietzek@leibniz-ipht.de

[b] N. Klosterhalfen, Prof. Dr. B. Dietzek-Ivanšić
Institute for Physical Chemistry (IPC)
Friedrich Schiller University Jena
Helmholtzweg 4, 07743 Jena, Germany

[c] N. Singh, Dr. A. Winter, Dr. M. Jäger, Prof. Dr. U.S. Schubert
Laboratory of Organic and Macromolecular Chemistry (IOMC)
Friedrich Schiller University Jena
Humboldtstr. 10, 07743 Jena, Germany
E-mail: ulrich.schubert@uni-jena.de

[d] N. Singh, Dr. A. Winter, Dr. M. Jäger, Prof. Dr. U.S. Schubert
Center for Energy and Environmental Chemistry Jena (CEEC Jena)
Friedrich Schiller University Jena
Philosophenweg 7a, 07743 Jena, Germany

[e] Dr. Phil Köhler
Institute for Inorganic and Analytical Chemistry (IAAC)
Friedrich Schiller University Jena
Humboldtstr. 8, 07743 Jena, Germany

Experimental Section

General Information. Unless stated otherwise, all reactions were performed using standard Schlenk techniques under a dry nitrogen atmosphere. Dry solvents were obtained from Merck or Across and used without further purification. Glassware was dried in an oven at 110 °C. NMR spectra were recorded at 25 °C on Bruker AVANCE I 300 MHz, AVANCE II 400 MHz, or AVANCE III 600 MHz instruments equipped with cryo-probeheads, using deuterated solvents from Euriso-Top. For ^{1}H NMR spectra, a line broadening of 0.3 was applied, and for ^{13}C NMR spectra, a line broadening of 1 was used. Chemical shifts are reported in ppm, referenced to the residual solvent signal. High-resolution electrospray ionization time-of-flight mass spectrometry (ESI-TOF MS) was conducted using a Bruker Daltonics MICROTOF II mass spectrometer. Microwave reactions were performed in an Initiator™ single mode microwave cavity at 2450 MHz (Biotage). All commercially available reagents were used as received unless otherwise noted. The solvents were received from commercial sources and were distilled for reactions and purifications. The precursor complex $Ru(DMSO)_4Cl_2$ was received commercially while the mono-diquinolylpyridine Ru(II) complex $[Ru(dqp)(CH_3CN)_3](PF_6)_2$ was

prepared following a developed procedure[2], and ligands were prepared adopting the literature protocol.[3] $Pd(dba)_2$ is bis(dibenzylideneacetone)palladium(0) and SPhos is 2-dicyclohexylphosphino-2′,6′-dimethoxybiphenyl. Chromatographic separations utilized silica gel (SiO2 60, 0.040–0.063 mm, Merck KGaA). Preparative thin layer chromatography (PTLC) was conducted using silica gel (SiO2 60 on 20 × 20 cm glass plates with a 1 mm layer thickness, Merck).

General procedure for the synthesis of ligands.[3] The ligands were prepared by following the procedure published via Suzuki coupling.[3] An overdried flask was initially loaded with Quinoline-8-yl boronic acid (4.6 mmol, 2.19 eq.), 2,6-dibromopyrazine or 2,6-dibromopyridine (2.1 mmol, 1 eq.), $Pd(dba)_2$ (0.038 g, 0.067 mmol, 0.032 eq.), SPhos (0.056 g, 0.133 mmol, 0.065 eq.), and potassium carbonate (1.372 g, 9.93 mmol, 4.73 eq.) as a base. This mixture was suspended in a solution consisting of degassed acetonitrile (25 mL) and water (5 mL). The system was evacuated and charged with N_2 and stirred at 110 °C overnight. Upon cooling to room temperature, water was added followed by extraction with CH_2Cl_2. The combined organic phases were concentrated under reduced pressure. Purification was achieved via column chromatography on silica gel using 5% methanol in dichloromethane as an eluent system collecting the bright fluorescent spot in UV-lamp as an off-white foamy solid of up to 55% yield.

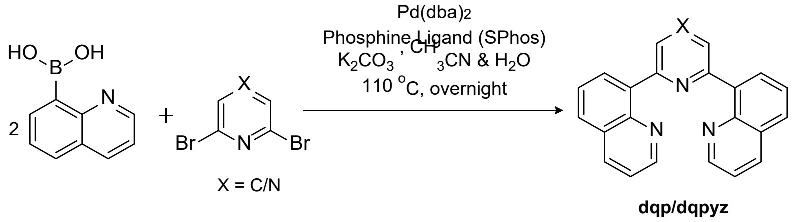

Scheme 1. Schematic representation of the synthetic routes to synthesize prospective ligands (dqp & dqpyz).

General procedure for the synthesis of ruthenium (II) homoleptic complexes.[4] A microwave vial was charged with the prepared ligand (0.29 mmol, 2 eq.), $RuCl_2(DMSO)_4$ (0.14 mmol, 1 eq.), and ethylene glycol (10 mL); sealed; and heated at 200 °C for 20 minutes using microwave irradiation. After cooling, the reaction mixture was diluted with an excess amount of water, and then subjected for the counter-anion exchange with an aqueous saturated solution of KPF_6 and dichloromethane. The organic phase was extracted with 5 to 10% acetonitrile in dichloromethane, collected fraction was dried over Na_2SO_4 and evaporated under rotary evaporator. The reduced amount was then directly subjected to a column chromatography on silica using $CH_3CN/H_2O/KNO_3$(sat) (40:4:1) as an eluent system. The chromatographic procedure was done twice, the main red band being collected in each case. The fractions containing the product were evaporated in vacuo until dryness and the residue was washed

with CH_3CN to remove excess KNO_3. CH_3CN was evaporated *in vacuo*, and the red solid was dissolved in the minimum amount of water for counter-anion exchange using a 3-fold excess of KPF_6, followed by filtration and washing of the formed precipitate with water and diethyl ether. The anion exchange was repeated once more using the minimum amount of water/acetone (10:1) to dissolve the solid. The product was dried under vacuum.

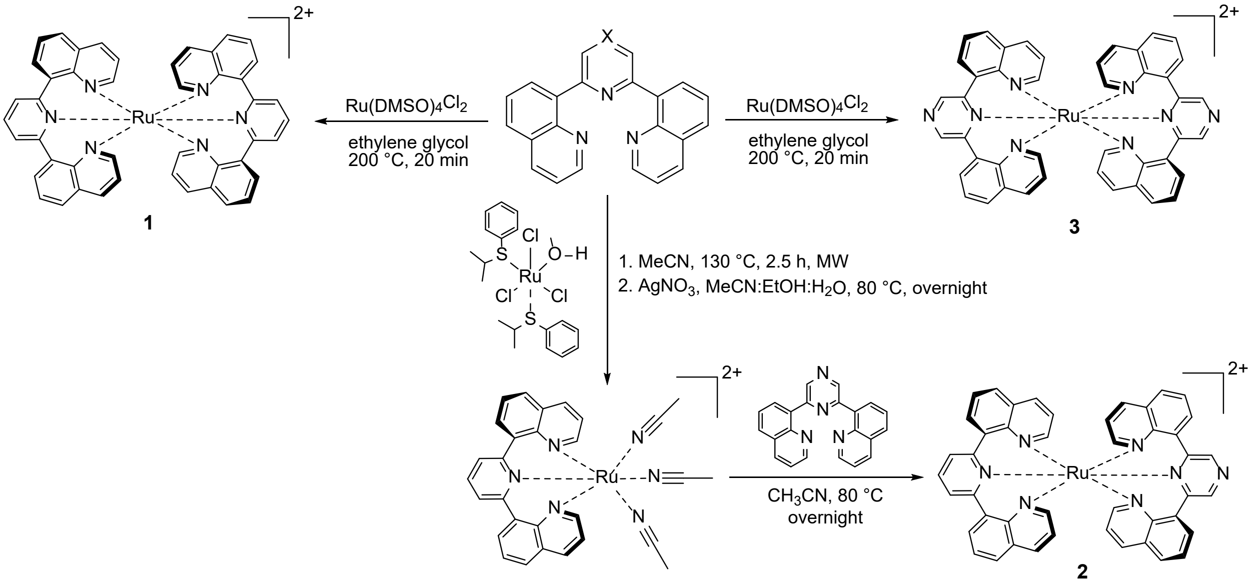

Scheme 2. Schematic representation of the stepwise synthetic routes to synthesize homoleptic and heteroleptic bistridentate Ru (II) complexes.

*2,6-Di(quinoline-8-yl) pyridine (**dqp**).* Followed the published procedure.[3]

*2,6-Di(quinoline-8-yl) pyrazine (**dqpyz**)*. Refer to the general procedure. The process afforded dqpyz ligand as an off-white foamy solid (0.286 g, 55% yield). ^1H NMR (300 MHz, $CDCl_3$): *δ* 9.39 ppm (2H, s, 2.8 Hz), 8.98 (2H, dd, J = 3.0, 2.6 Hz), 8.29 (4H, dd, J = 9.0, 2.4 Hz), 7.88 (2H, dd, J = 9.0, 2.3 Hz), 7.68 (2H, t, J = 6.0, 2.3 Hz), 7.44 (2H, m, J = 3.0, 2.2 Hz). ^{13}C NMR (100 MHz, CD_3CN): δ 151.8, 150.6, 145.7, 136.5, 136.3, 131.8, 129.4, 128.6, 126.8, 121.4. ESI-MS: calcd for $C_{23}H_{15}N_3$ 333.13 au, found [M+H] = 335.0 m/z.

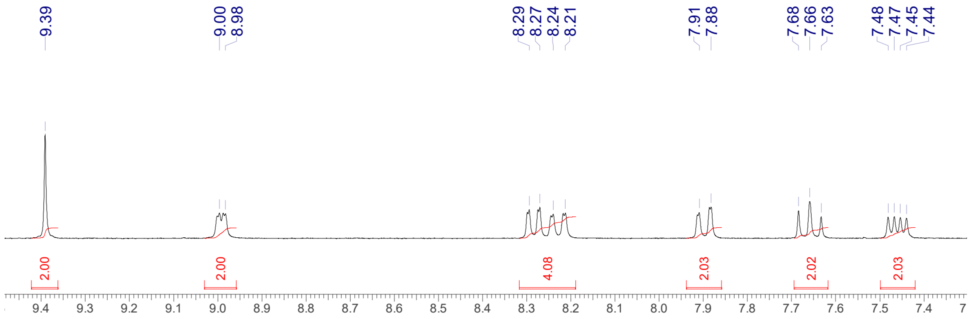

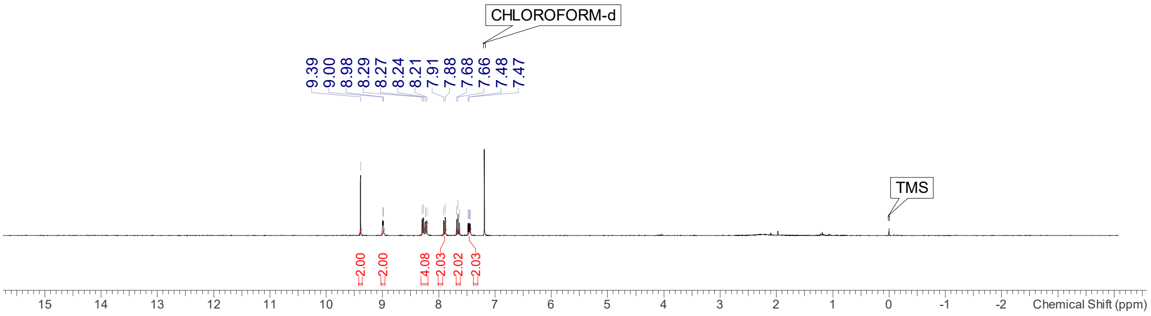

Figure S1. ^{1}H-NMR (300 MHz, $CDCl_3$): 2,6-bis(quinoline-8-yl) pyrazine (**dqpyz**).

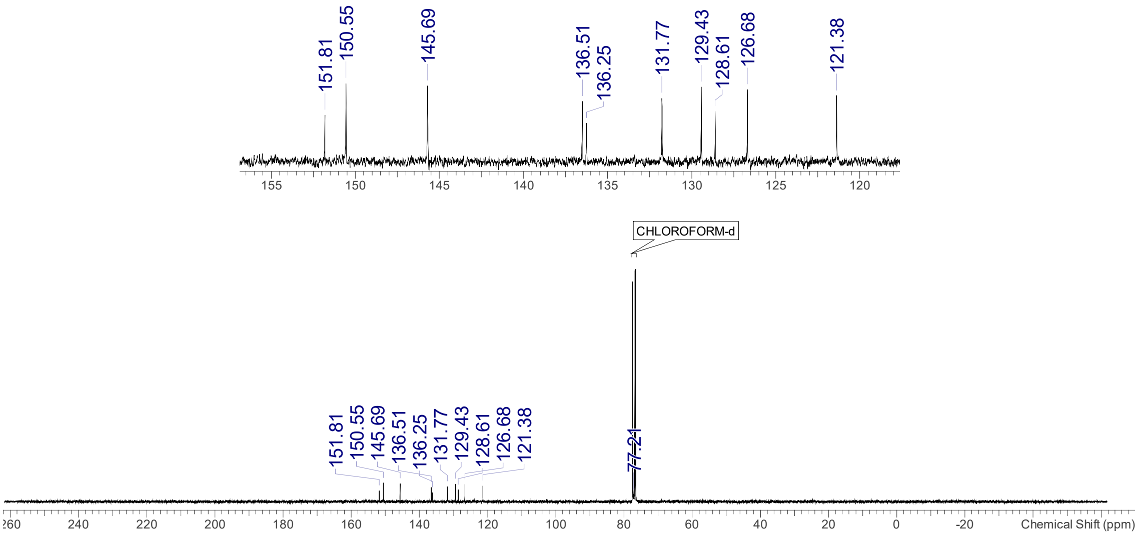

Figure S2. ^{13}C-NMR (150 MHz, $CDCl_3$): 2,6-bis(quinoline-8-yl) pyrazine **(dqpyz)**.

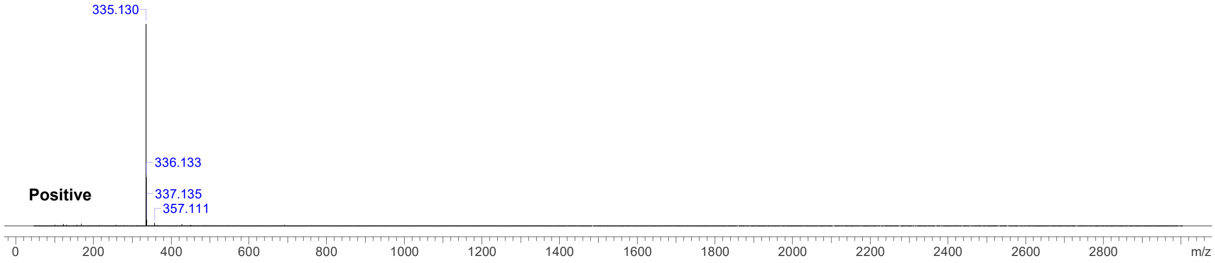

Figure S3. ESI-TOF-MS of **dqpyz** Ligand.

Complex Ru(dqp)$_2$(PF$_6$)$_2$. Followed the published procedure.[4]

Complex Ru(dqpyz)$_2$(PF$_6$)$_2$. Yield: 18.7 mg, 22%. ^{1}H NMR (300 MHz, CD_3CN): δ 9.00 ppm (4H, s, 2.7 Hz), 8.15 (4H, dd, J = 9.0, 2.4 Hz), 8.06 (4H, dd, J = 6.0, 2.4 Hz), 7.90 (4H, dd, J = 9.0, 2.3 Hz), 7.80 (4H, dd, J = 9.0, 2.3 Hz), 7.55 (4H, m, J = 9,0, 2.2 Hz) 7.14 (4H, m, J = 3.0, 2.1 Hz). ^{13}C NMR(100 MHz, CD_3CN): δ 159.1, 151.4, 148.9, 139.0, 133.9, 132.1, 129.4, 127.6, 123.1. HRMS-ESI: calculated for $C_{44}H_{28}F_{12}N_8P_2Ru$ 1059.56 au, found $[M - 2PF_6^-]^{2+}$ 385.07

m/z. Single crystals suited X-ray diffraction analysis were obtained from vapor diffusion of Et_2O into a CH_3CN solution.

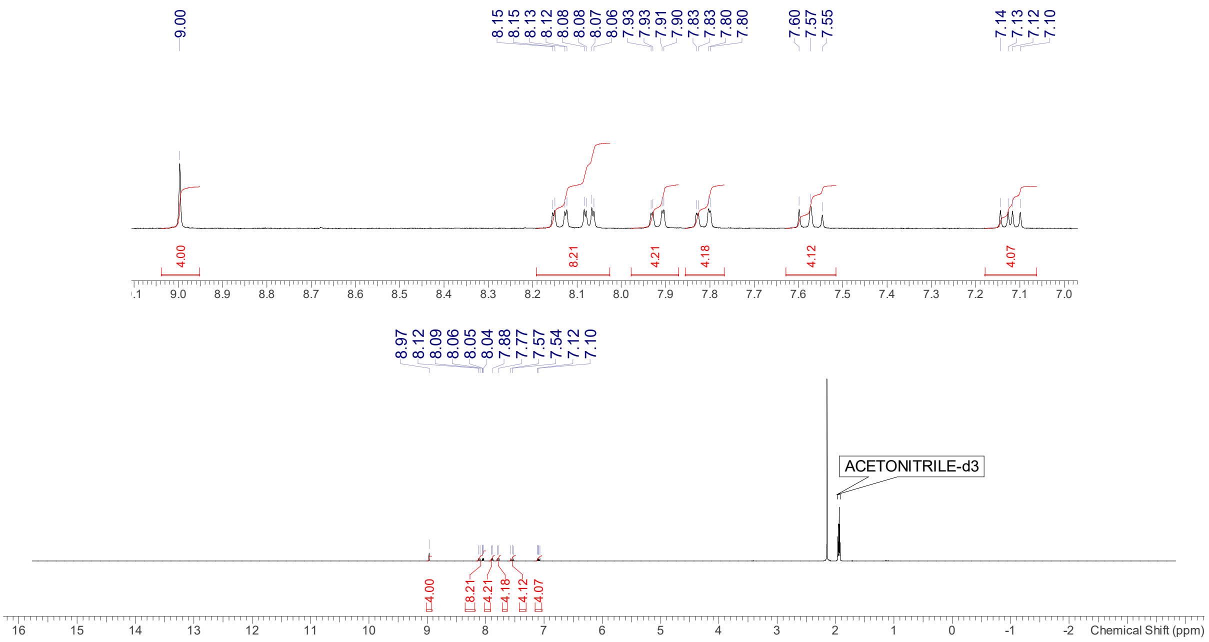

Figure S4. ^{1}H-NMR (300 MHz, CD_3CN): **Complex 3** [Ru(dqpyz)$_2$] (PF_6)$_2$.

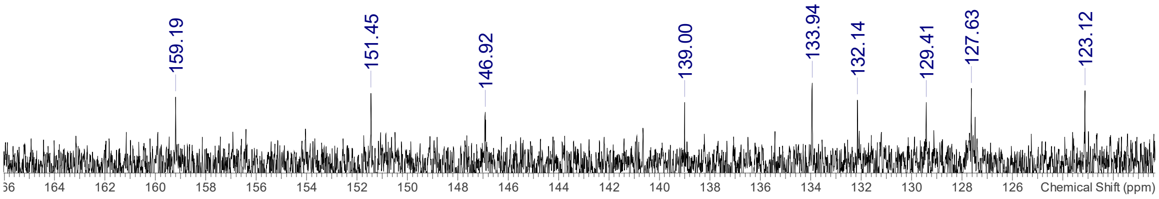

Figure S5. *^{13}C-NMR (100 MHz, CD_3CN): **Complex 3** [Ru(dqpyz)$_2$] (PF_6)$_2$*

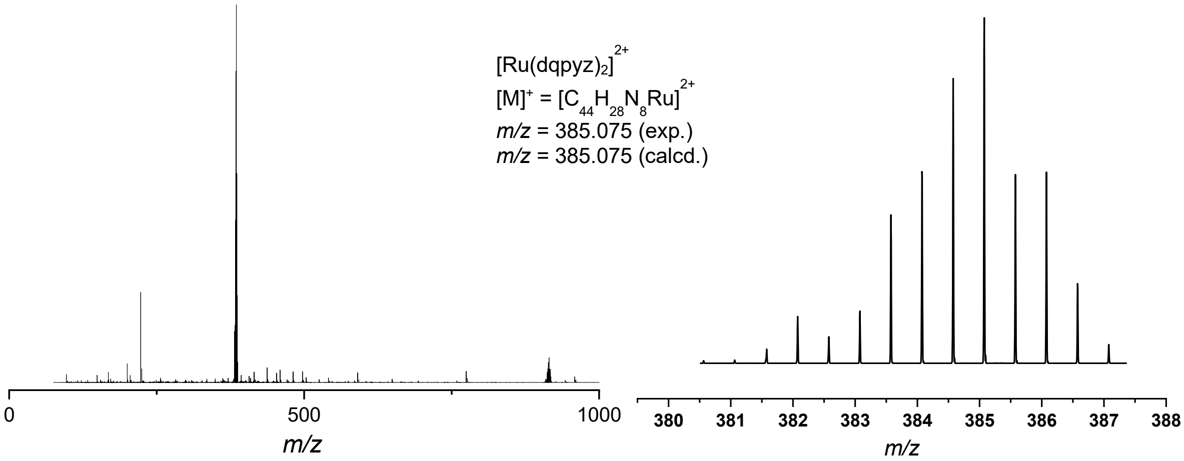

Figure S6. HRMS-ESI of Ru(dqpyz)$_2$(PF_6)$_2$ complex.

Complex Ru(dqp)(dqpyz)(PF$_6$)$_2$. A microwave vial was charged with dqpyz (0.018g, 0.057 mmol, 1 eq.), [Ru(dqp)(CH$_3$CN)$_3$(PF$_6$)$_2$] [3](0.030 g, 0.053 mmol, 1 eq.), and acetonitrile (6 mL); sealed; and heated at 80 °C overnight. The purification was done as described for Ru(dqpyz)$_2$(PF$_6$)$_2$. Yield 22 mg, 18%. ^{1}H NMR (300 MHz, CD$_3$CN): *δ* 8.93 ppm (3H, dd, J = 9.0, 6 Hz), 8.43 (3H, dd, J = 9.0, 9 Hz), 8.27 (3H, dd, J = 9.0, 9 Hz), 8.15 (3H, m, J = 9.0, 6 Hz), 8.06 (6H, m, J = 9.0, 6 Hz), 7.85 (1H, d, J = 3 Hz), 7.76 (3H, t, J = 6 Hz), 7.68 (2H, t, J = 3 Hz), 7.58 (3H, q, J = 3.0 Hz), 7.44 (1H, t, J = 6Hz), 7.03(1H, q, J = 3 Hz). ^{13}C NMR (100 MHz, CD$_3$CN): δ 156.5, 150.8, 145.9, 137.6, 133.5, 131.7, 129.9, 129.3, 128.4, 127.3, 127.1, 125.8, 122.5, 122.1. HRMS-ESI: calculated for $C_{45}H_{29}F_{12}N_7P_2Ru$ 1058.56 au, found [M − 2PF6$^-$]$^{2+}$ = 384.50 m/z. Single crystals suited for X-ray diffraction analysis were obtained from vapor diffusion of Et$_2$O into a CH$_3$CN solution.

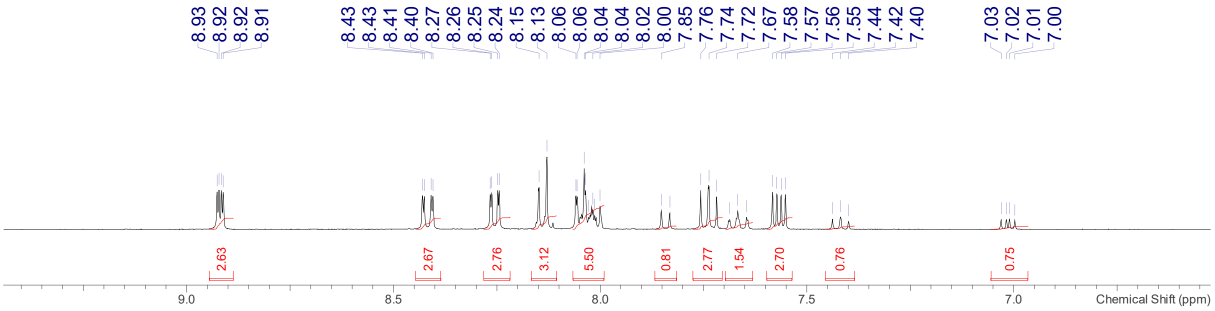

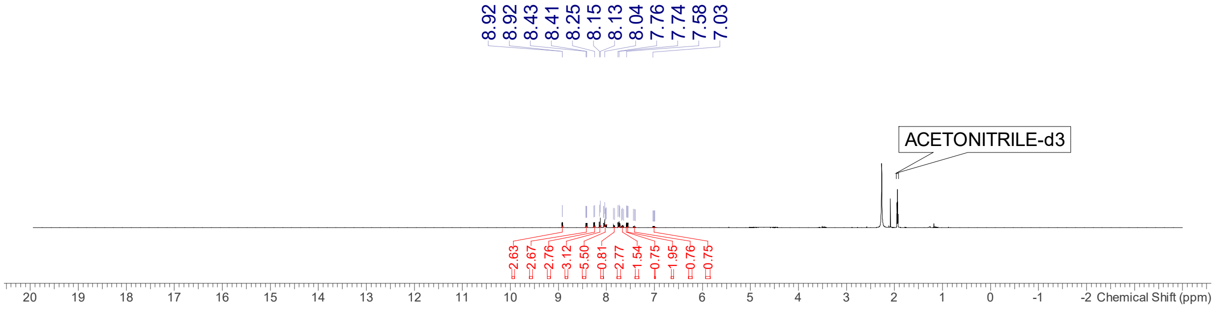

Figure S7. ^{1}H-NMR (300MHz, CD$_3$CN): [Ru(dqp)(dqpyz)] (PF$_6$)$_2$ (Mer-isomer).

Figure S8. ^{13}C-NMR (100MHz, CD$_3$CN): [Ru(dqp)(dqpyz)] (PF$_6$)$_2$.

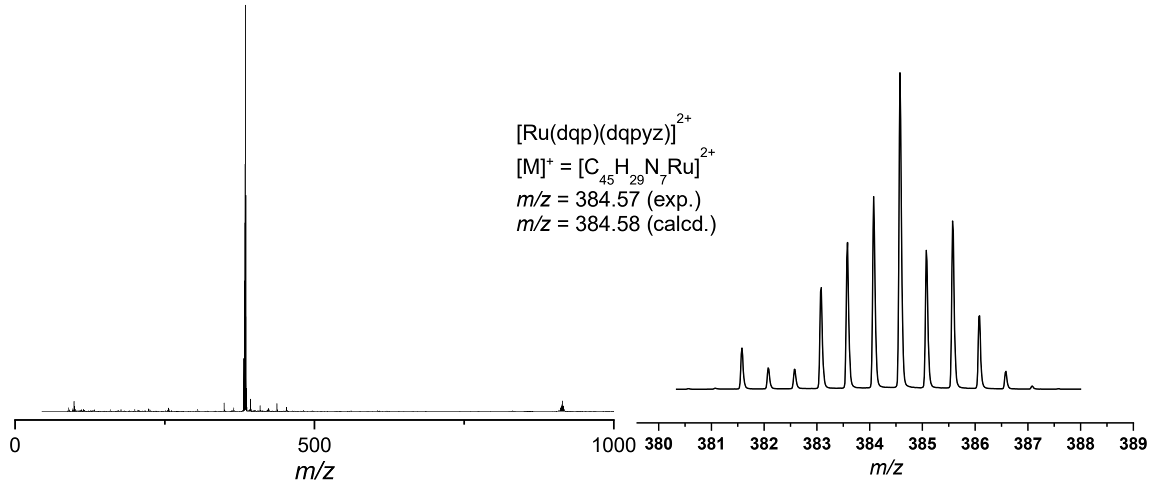

Figure S9. HRMS-ESI of Ru(dqp)(dqpyz)(PF_6)$_2$.

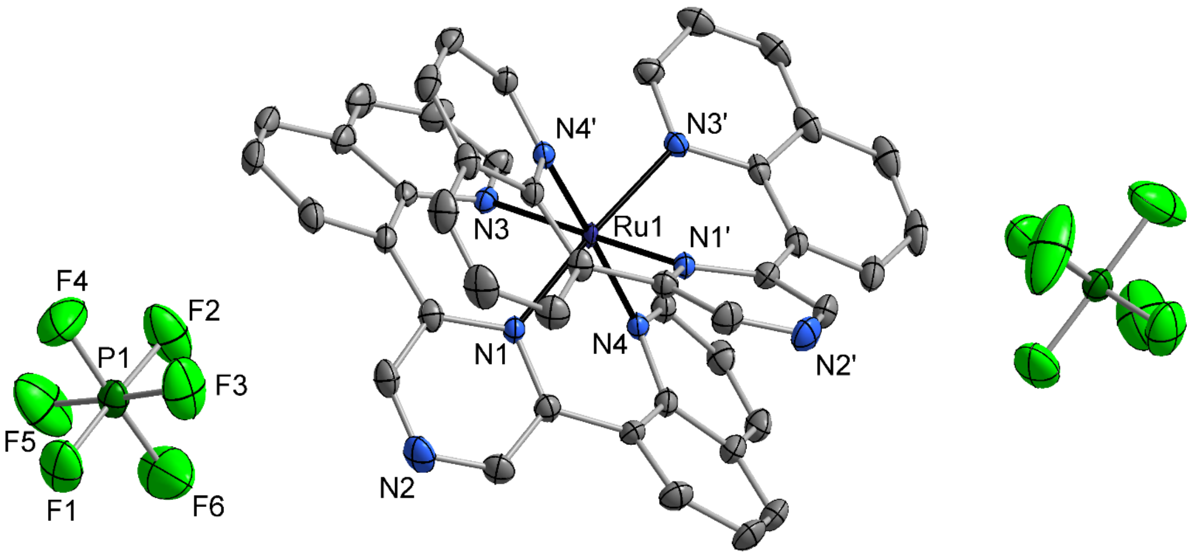

Figure S10. Molecular structure of [Ru(dqpyz)$_2$](PF_6)$_2$ (**3**) ·2 MeCN in the crystal, showing the atom numbering scheme. Displacement ellipsoids are drawn at the 50% probability level, hydrogen atoms and solvent of crystallization omitted for clarity.

Table S1. Crystal data and details on X-ray structure refinement for compound **3**.

**Compound**	**[Ru(dqpyz)$_2$](PF$_6$)$_2$ ·2 CH$_3$CN**
CCDC deposition number	2377177
Molecular formula sum	$C_{48}H_{34}F_{12}N_{10}P_2Ru$
Formula weight / g mol^{-1}	1141.86
Crystal system	monoclinic
Space group	C2/c
Cell metric a / Å	12.486(1)
b / Å	25.614(3)
c / Å	15.011(2)
α / deg.	90
β / deg.	114.092(3)
γ / deg.	90
Cell volume / Å^3	4382.8(8)
Molecules per cell z	4
Electrons per cell F_{000}	2296
Calcd. density ρ / g cm^{-3}	1.730
μ / mm^{-1} (Mo-K$_α$)	0.534
Crystal shape and color	red plate
Crystal size / mm	0.24×0.15×0.09
θ range / deg.	1.966 … 27.103
Reflections collected	24689
Reflections unique	4835
Reflections with I>2σ(I)	4068
Completeness of dataset	99.9 %
R_{int}	0.0437
Parameters; Restraints	331; 9
R_1 (all data, I>2σ(I))	0.0721; 0.0566
wR_2 (all data, I>2σ(I))	0.1384; 0.1292
GooF (F^2)	1.102
Max. residual peaks	–0.683; 1.614

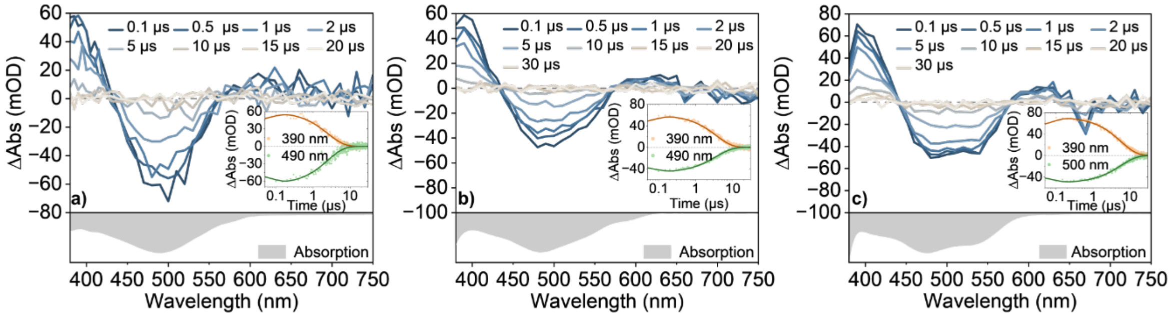

Figure S11. Nanosecond transient absorption spectra of complexes **1-3** in oxygen-free MeCN sorted in order a-c. The corresponding absorption spectra in the same solvent are plotted (and inverted) in the bottom panel. For all measurements 20 ns excitation pulses centered around 355 nm were used. In the inset, the corresponding kinetics at the indicated wavelengths are shown. Experimental data points are shown as dots and the results of single exponential (global) fits are plotted as solid lines. The fitted lifetimes of 2.5, 3.6, and 5.6 μs match very well with the time-resolved emission traces in the main-text.

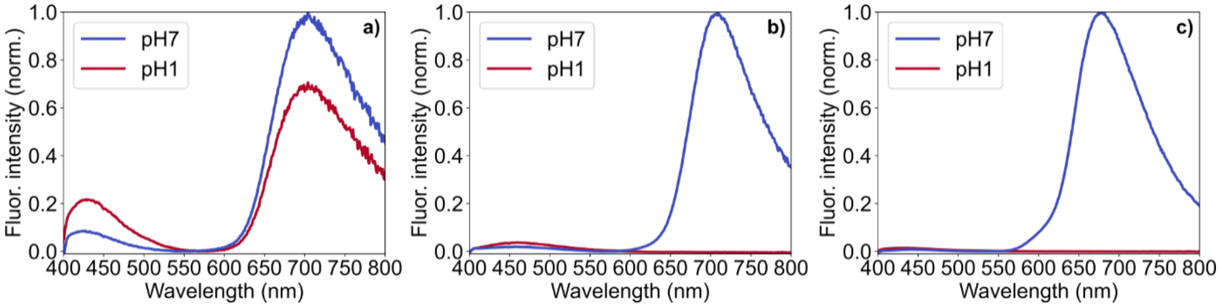

Figure S12. Emission spectra of complexes **1-3** in aqueous solutions of pH 7 and pH 1 (0.1 M H_2SO_4) after excitation at 350 nm sorted in order a-c. For both complexes with a pyrazine unit the MLCT emission is seen to disappear as a consequence of ESPT.

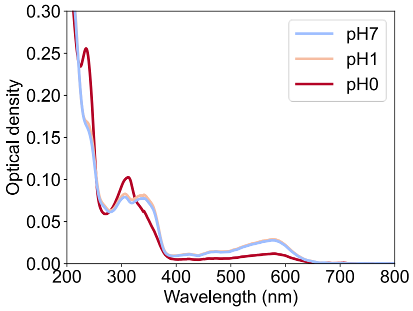

Figure S13. Absorption spectra of complex **1** in aqueous solutions of varying pH value. No differences are seen between the measurements at pH 7 and 1. The presence of unbound ligands is therefore ruled out. Reducing the pH even further down to 0 results in degradation of the complex which is accompanied by an increase of the (free) ligand absorption and a disappearing MLCT transition.

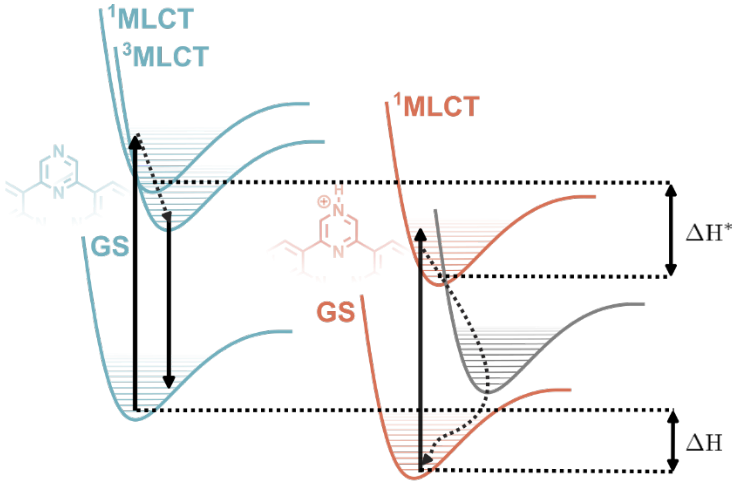

$$pKa^* - pKa = \frac{\log(e)hc}{k_bT}(\tilde{\nu}_{00}^{base} - \tilde{\nu}_{00}^{acid})$$

Figure S14. Förster cycle analysis for the ground- and excited-state protonation process of complexes **2** and **3**. In order to determine the $\upsilon_{0\to0}$ transition energies, an average of both the absorption and emission maxima is usually taken. Due to the nonradiative decay pathway of the protonated form, this type of analysis is not possible here and we resort to using only the absorption spectra to estimate the ΔpKa value. These estimations are later corroborated by fluorescence titration experiments. Adapted from [5].

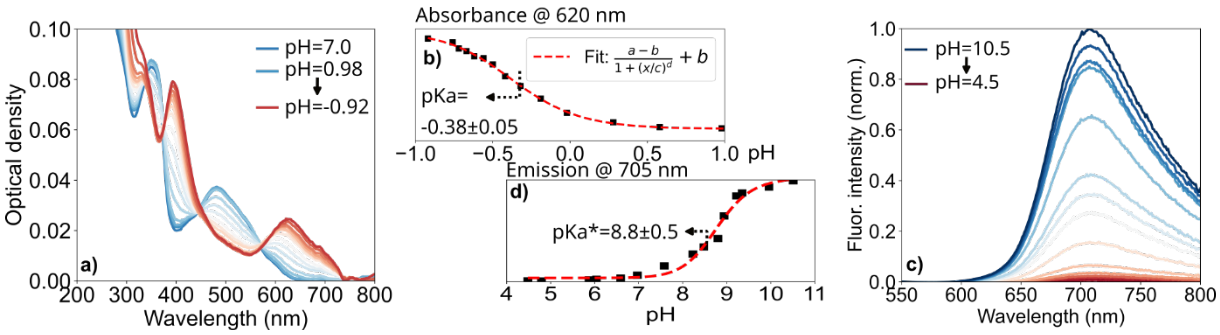

Figure S15. a) Spectrophotometric titration of complex **2** with H_2SO_4. b) Cross section of the data set in a) at 620 nm and logistic fit. The whole data set was shifted by 5 pH units. Since for complex **2** only single protonation is possible, all data points were considered for the fit. At pH -1.2 degradation of the complex was seen. The pKa* and respective error are averaged over the wavelength range 600-680 nm. The theoretical pH-values are calculated assuming that H_2SO_4 dissociates one of its hydrogen atoms and the absolute absorbance values are concentration corrected. c) Fluorescence spectra in different pH buffer solutions after excitation at 350 nm. d) Fluorescence intensities at 705 nm with the same logistic fit used in b). The equivalence point is calculated as the average between 680 and 780 nm. The measurement error is determined by the accuracy of the used pH-meter.

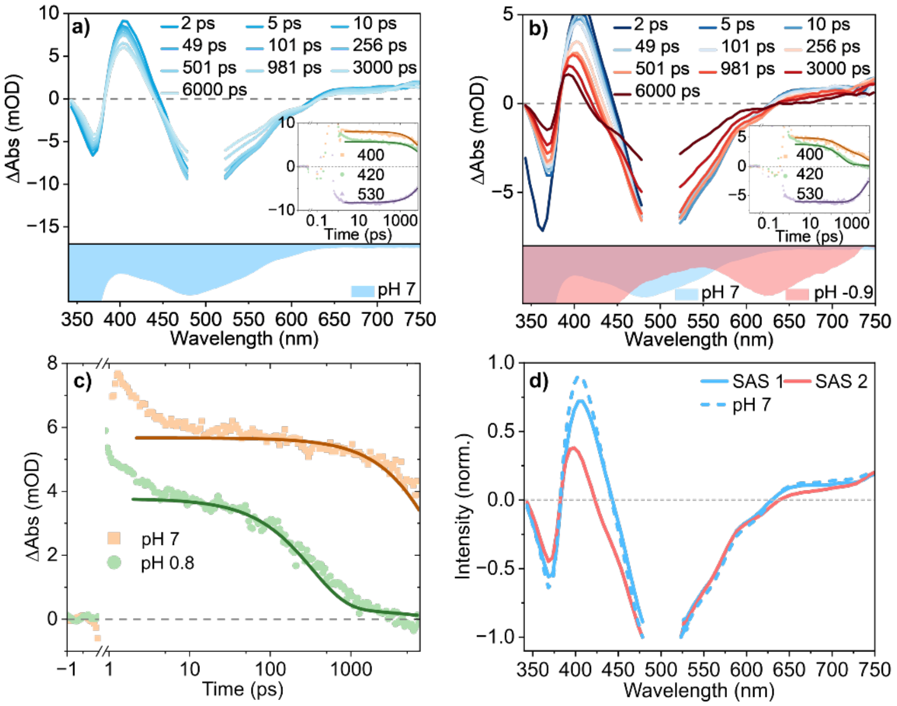

Figure S16 . Transient absorption data of complex **2** in aqueous solutions of pH 7 (a) and pH 0.8 (b) acidified with H_2SO_4 following MLCT excitation (λ_{exc}=490 nm). c) elucidates the differences in the kinetics at pH 7 and 0.8 due to ESPT at 420 nm. In all cases only values above 2 ps were considered for the global fit which yields the (normalized) SAS in d) for the measurement at pH 0.8. SAS1 is in good agreement with the measurement of complex **2** at pH 7.

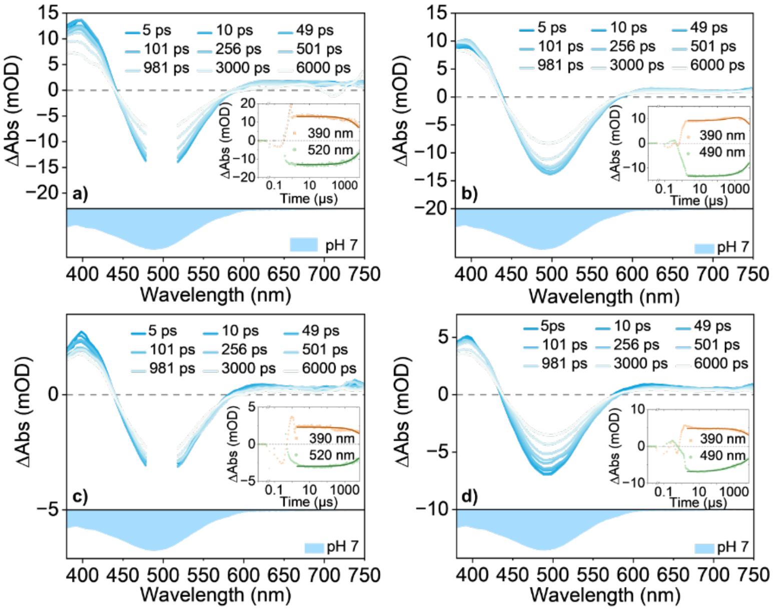

Figure S17. Transient absorption spectra of complex **1** in solutions of pH 7 (a) and b)) and pH 0.8 (c) and d)) after excitation at the MLCT band (left, λ_{exc}=490 nm) and at the ligand band (right, λ_{exc}=350 nm). No differences are seen between the different pH values after MLCT excitation since ESPT is not possible for this complex. For the ligand-based excitation on the other hand, the kinetics seem to be different. While for pH 0.8 only one decay channel seems to be accessible, two sequential steps are seen for pH 7. For all data sets only population times greater than 2 ps were considered for the fit. The corresponding absorption spectra at pH7 are shown in the bottom panel and individual kinetic traces are presented in the inset along with a global fit. In all cases but pH 7 / λ_{exc}=350 nm a single exponential model was used while the latter required two sequential components.

Preliminary photocatalysis experiments

In order to test the practical applicability of our complexes as photocatalytic bases, we added $[Ru(dqpyz)_2]^{2+}$ (5 mM) to a solution of benzaldehyde (50 mM) and nitroethane (500 mM) in hexafluoroisopropanol (HFIP). The expected nitro-aldol reaction that follows is in last consequence the result of the deprotonation of nitroethane in the presence of the photobasic metal complexes. To trigger this reaction, the solution was continuously illuminated with 420 nm LED light. The proton in nitroethane's α-position is allegedly abstracted either by the photobasic complex itself or by the alkoxide (formed by a preceding deprotonation of HFIP by the complex). The thusly formed carbanion is expected to perform the nucleophilic attack on benzaldehyde. This reaction was tracked by IR spectroscopy *via* the decreasing intensity of the ν(C=O) band of benzaldehyde at 1695 cm^{-1}. Octanol (64 mM) was added to the reaction solution as an internal standard due to its low volatility and high pKa. Since the latter does not interfere in the condensation reaction, all spectra were subsequently normalized to its CH vibrations in the range 2700 to 3100 cm^{-1}. Figure S18a displays ATR-FTIR spectra that were recorded from the reaction solution under 420-nm irradiation in intervals of 1 h. The integrals of the carbonyl stretch bands (following baseline correction) are displayed on the right side and provide a metric to follow the reaction progress. After 45 h the intensity of the ν(C=O) band is seen to decrease. On the contrary, without illumination (control, Figure S18b) the IR intensity of the C=O vibration is still quite prominent even after 45 h. These results indicate that the reaction proceeds but do not explain the initial decrease of the ν(C=O) integral in the non-illuminated case. Furthermore, the possibility of benzaldehyde degradation upon illumination cannot be excluded by this method.

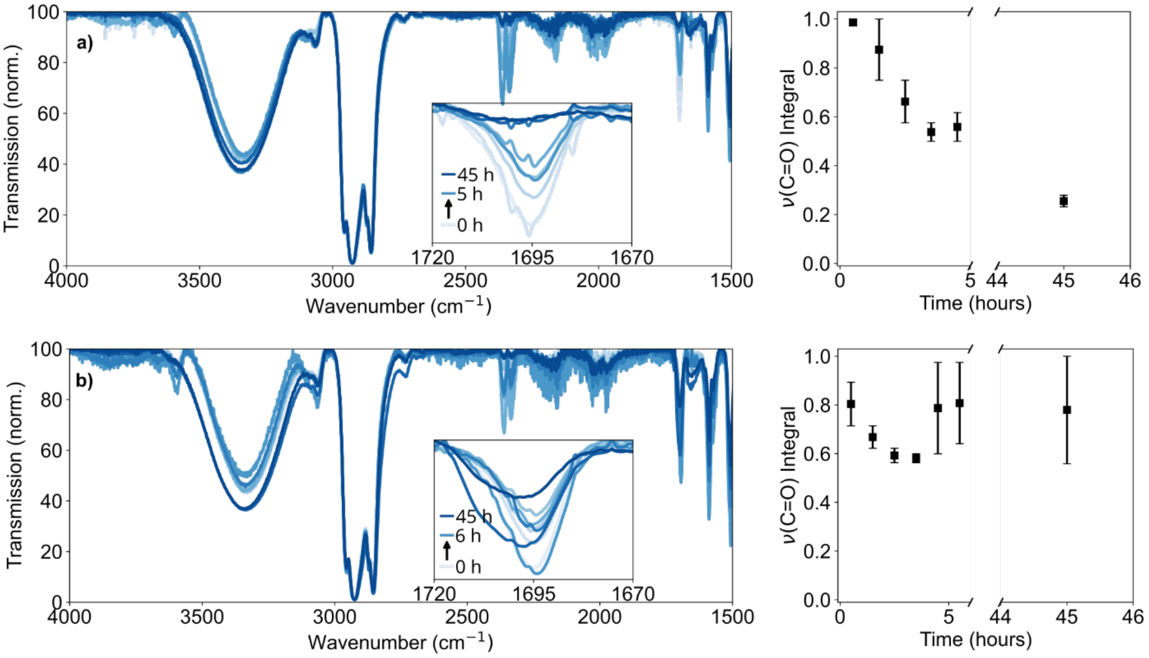

Figure S18. ATR-FTIR spectra recorded for the reaction mixture of benzaldehyde, nitroethane, octanol and complex **3** in HFIP over the course of several hours with a) and without b) illumination by a 420 nm LED. All spectra are normalized to the C-H vibrations of octanol in the range 2700 – 3000 cm^{-1}.

During the review process we have addressed the aforementioned concerns and have repeated the photocatalysis experiments in order to include the following experiments and controls:

- $Ru(dqpyz)_2$ + Nitroethane + Benzaldehyde (under ilumination)
- $Ru(dqp)_2$ + Nitroethane + Benzaldehyde (under ilumination)
- Nitroethane + Benzaldehyde (under ilumination)
- $Ru(dqpyz)_2$ + Nitroethane + Benzaldehyde (no illumination)

In all cases a catalyst concentration of 5 mM was used to match the previously performed experiments. All remaining concentrations were also kept constant. Following a 24-hour reaction period, the mixtures were cooled to room temperature, and 20 μL aliquots were extracted for NMR analysis. Mesitylene was added as an internal standard which is characterized by a well-resolved singlet at 2.47 ppm and at 7.0 ppm corresponding to 9 and 3 protons respectively. The integral of the former peak was consistently normalized to 9.0 across all spectra. This allowed us to quantify the amount of product formed in the different reactions. The recorded ^{1}H and ^{13}C-NMR spectra are presented below.

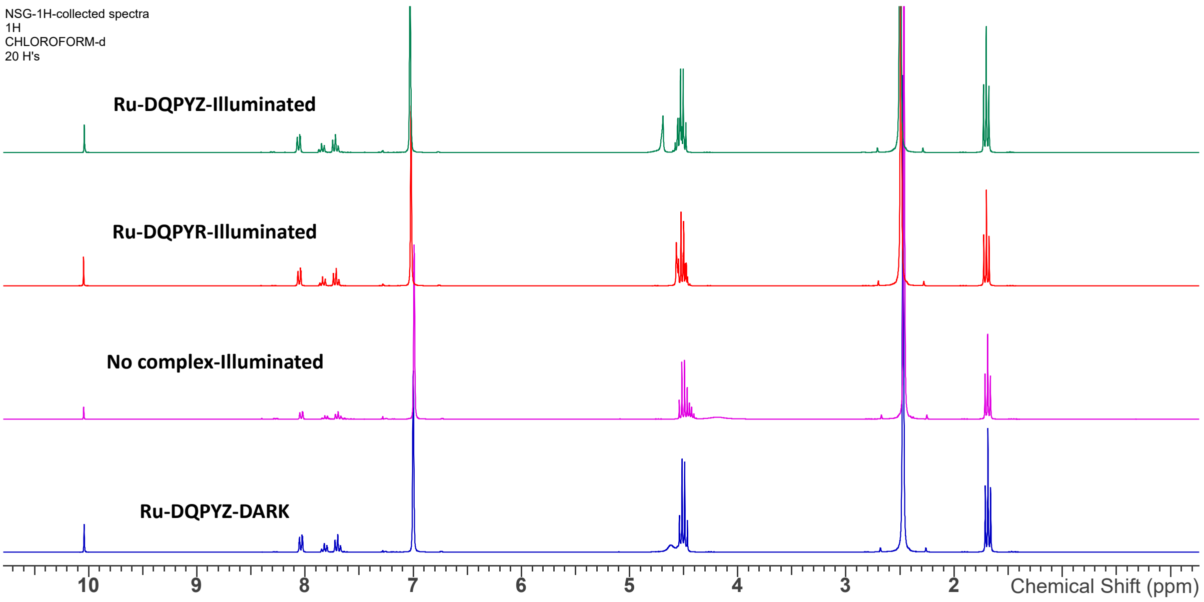

Figure S19. Collected ^{1}H-NMR (300MHz, CDCl3) spectra.

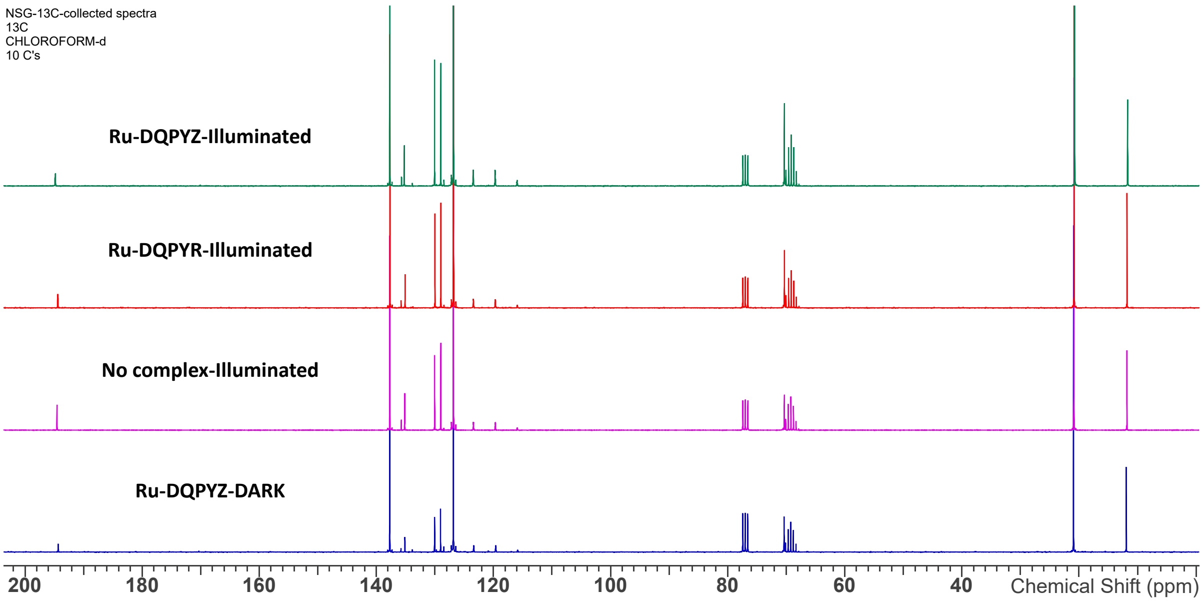

Figure S20. Collected ^{13}C-NMR (100MHz, CDCl3) spectra.

The NMR analysis indicates that the anticipated β-nitroalcohol products, 2-nitro-1-phenylprop-2-en-1-ol and 2-nitro-1-phenylpropan-1-ol, were either not formed at all or not in quantities that allow for their detection. It is striking that the illuminated reaction mixture with Ru(dqpyz)$_2$ shows an additional peak at 4.69 ppm but its assignment is not clear since no other new peaks were detected in the ^{1}H- and ^{13}C-NMR spectra. Furthermore, quantification of the concentrations of Benzaldehyde and Nitroethane, with respect to their initial concentrations, revealed that the above-mentioned reaction mixture contains the lowest residual concentration of the reactants. This might be an indication of an ongoing reaction (see Table S2). Nevertheless, a reduction of the reactant concentrations is also seen for the control experiments which indicates that these degrade with time.

Table S2. Relative concentrations in percent of the reactants after 24 hours.

Experiment	Benzaldehyde (%)	Nitroethane (%)
Ru-DQPYZ-Illuminated	52.08	88.63
Ru-DQPYR-Illuminated	72.60	92.31
No complex-Illuminated	89.42	98.84
Ru-DQPYZ-DARK	63.60	97.51

Overall, both the ATR-FTIR as well as the ^{1}H-NMR experiments indicate that a reaction might be taking place. Unfortunately, the reaction products are not formed in sufficient yields to allow

for their characterization. We speculate that this is due to the pKa of nitroethane being on the same order of magnitude as the pKa* of $[Ru(dqpyz)_2]^{2+}$, *i.e.* 8.5 and 8.6±0.5, respectively.

References:

[1] I. P. Evans, A. Spencer, G. Wilkinson, J. Chem. Soc., Dalton Trans. **1973**, 204-209.

[2] M. Jäger, A. Smeigh, F. Lombeck, H. Görls, J.-P. Collin, J. P. Sauvage, L. Hammarström, O. Johansson, *Inorg. Chem.* **2009**, *49*, 374-376.

[3] M. Abrahamsson M. Jäger R. J. Kumar T. Osterman P. Persson H. C. Becker O. Johansson, L. Hammarström*, J. Am. Chem. Soc.* **2008**, *130*, 15533-15542.

[4] M. Jäger, R. J. Kumar, H. Görls, J. Bergquist, O. Johansson, *Inorg. Chem.* **2009**, *48*, 3228-3238.

[5] J. F. Ireland, P. A. H. Wyatt, in *Adv. Phys. Org. Chem.* (Ed.: V. Gold), Academic Press, London, **1976**
